# Diagnosis of physical and mental health conditions in primary care during the COVID-19 pandemic: a retrospective cohort study

**DOI:** 10.1016/S2468-2667(20)30201-2

**Published:** 2020-09-23

**Authors:** Richard Williams, David A Jenkins, Darren M Ashcroft, Ben Brown, Stephen Campbell, Matthew J Carr, Sudeh Cheraghi-sohi, Navneet Kapur, Owain Thomas, Roger T Webb, Niels Peek

**Affiliations:** a The National Institute for Health Research Greater Manchester Patient Safety Translational Research Centre, University of Manchester, Manchester, UK; b Division of Informatics, Imaging and Data Science, University of Manchester, Manchester, UK; c Division of Pharmacy and Optometry, University of Manchester, Manchester, UK; d Division of Population Health, Health Services Research and Primary Care, University of Manchester, Manchester, UK; e Division of Psychology and Mental Health, University of Manchester, Manchester, UK; f Greater Manchester Mental Health NHS Foundation Trust, Manchester, UK; g Langworthy Medical Practice, Salford, UK

## Abstract

**Background:**

To date, research on the indirect impact of the COVID-19 pandemic on the health of the population and the health-care system is scarce. We aimed to investigate the indirect effect of the COVID-19 pandemic on general practice health-care usage, and the subsequent diagnoses of common physical and mental health conditions in a deprived UK population.

**Methods:**

We did a retrospective cohort study using routinely collected primary care data that was recorded in the Salford Integrated Record between Jan 1, 2010, and May 31, 2020. We extracted the weekly number of clinical codes entered into patient records overall, and for six high-level categories: symptoms and observations, diagnoses, prescriptions, operations and procedures, laboratory tests, and other diagnostic procedures. Negative binomial regression models were applied to monthly counts of first diagnoses of common conditions (common mental health problems, cardiovascular and cerebrovascular disease, type 2 diabetes, and cancer), and corresponding first prescriptions of medications indicative of these conditions. We used these models to predict the expected numbers of first diagnoses and first prescriptions between March 1 and May 31, 2020, which were then compared with the observed numbers for the same time period.

**Findings:**

Between March 1 and May 31, 2020, 1073 first diagnoses of common mental health problems were reported compared with 2147 expected cases (95% CI 1821 to 2489) based on preceding years, representing a 50·0% reduction (95% CI 41·1 to 56·9). Compared with expected numbers, 456 fewer diagnoses of circulatory system diseases (43·3% reduction, 95% CI 29·6 to 53·5), and 135 fewer type 2 diabetes diagnoses (49·0% reduction, 23·8 to 63·1) were observed. The number of first prescriptions of associated medications was also lower than expected for the same time period. However, the gap between observed and expected cancer diagnoses (31 fewer; 16·0% reduction, −18·1 to 36·6) during this time period was not statistically significant.

**Interpretation:**

In this deprived urban population, diagnoses of common conditions decreased substantially between March and May 2020, suggesting a large number of patients have undiagnosed conditions. A rebound in future workload could be imminent as COVID-19 restrictions ease and patients with undiagnosed conditions or delayed diagnosis present to primary and secondary health-care services. Such services should prioritise the diagnosis and treatment of these patients to mitigate potential indirect harms to protect public health.

**Funding:**

National Institute of Health Research.

## Introduction

Since the start of the COVID-19 pandemic, many countries have imposed stringent restrictions on the movement and interaction of populations; commonly known as lockdown. In the UK, the earliest case of COVID-19 was confirmed on Feb 21, 2020,^1^ and the first death associated with COVID-19 occurred on March 5, 2020.^2^ On March 16, 2020, the UK Government suggested that non-essential travel should be avoided and on March 20, 2020, sites such as restaurants and gyms were closed. A nationwide lockdown was implemented on March 23, 2020, which required people to stay at home, leaving only for limited purposes (one form of exercise per day, shopping for necessities, any medical need, essential work).

The COVID-19 pandemic and associated public health emergency is likely to have affected patients and the health service with regard to non-COVID conditions. People might have been unwilling to attend a health-care facility because of concerns about catching COVID-19, or due to the misconception that the National Health Service (NHS) was only available for patients with COVID-19. Emergency department attendance declined by 25% in the week after lockdown was implemented,^3^ and data from the Royal College of General Practitioners surveillance system^4^ has shown that weekly reported incidence of asthma, intestinal infectious diseases, upper respiratory tract infections, and acute respiratory tract infections was markedly reduced. Substantial reductions in hospital admissions for suspected heart attacks^5^ and strokes,^6^ and urgent general practitioner (GP) referrals for cancer^7^, ^8^ have also been reported.

**Research in context**

**Evidence before this study**

We searched PubMed from database inception to June 18, 2020, for articles published in English, with titles that included the search terms (“covid*” or “coronavirus” or “sars-cov-2”), and title or abstracts that included the search terms (“indirect impact” or “missed diagnos*” or “missing diagnos*” or “delayed diagnos*” or ((“present*” or “consult*” or “engag*” or “access*”) AND (“reduction” or “decrease” or “decline”)). Emerging evidence suggests that many patients have not engaged with health-care services during the COVID-19 public health emergency. This reduction has led to decreases in the number of patients presenting with some acute conditions, such as myocardial infarctions and strokes, and a reduction in the number of diagnoses of some chronic conditions, such as asthma and cancer. However, the full extent of potential missed diagnoses has not yet been quantified.

**Added value of this study**

To our knowledge, this is the first population-based study to assess and quantify the indirect impact of the COVID-19 emergency on potential missed diagnoses in primary care. In this study, we used the electronic health records for a socioeconomically deprived urban population of approximately a quarter of a million people, to investigate the indirect impact of COVID-19 on UK primary care. We have shown that, for many common conditions, a significant reduction in the incidence of initial diagnoses has been observed since the start of the pandemic. These reductions are likely to represent a substantial increase in missed diagnoses. We corroborated the results by investigating the first prescriptions of medications highly predictive of a new diagnosis of the conditions we assessed.

**Implications of all the available evidence**

Primary and secondary health-care services, and specialist mental health services and counselling services, should prepare for an increase in demand once the initial acute phase of the pandemic recedes, and take steps to prioritise patients with delayed diagnoses. Further research should focus on the impact of missed diagnoses in terms of excess morbidity and mortality due to the COVID-19 pandemic and the potential for worse outcomes among patients with delayed diagnoses.

Other factors might have affected primary health-care attendance and hospital admissions. The widespread shift in UK primary care to remote consultations might have affected how clinicians do consultations, and patients without the ability to participate in video consultations (ie, those with no access to a smartphone, computer, or the internet) might not have received any care. The NHS postponed most elective operations in March, 2020, and diagnostic capacity might have been reduced with laboratories focusing on COVID-19 tests. Thus, it is important to quantify the extent of the indirect impact of COVID-19 on the diagnosis and treatment of non-COVID conditions, particularly conditions with relatively high prevalence, because they represent a major burden on general practices and the community, and conditions of sufficient severity that missed or delayed diagnoses could potentially have a clinically significant effect on an individual's long-term health and mortality risk.

Salford is a metropolitan borough of Greater Manchester (UK) with a population of approximately 250 000 people. Of 317 areas in England, Salford is the eighteenth most deprived,^9^ with the twentieth highest level of age-standardised mortality.^10^ Between March 1 and May 31, 2020, Salford had the fourth highest age-standardised all-cause mortality rate in England and Wales and the third highest age-standardised COVID-19 mortality rate outside of London.^11^

The full extent of potential missed diagnoses due to the COVID-19 emergency has not yet been quantified. The aim of our study was to investigate the indirect impact of the COVID-19 public health emergency on general practice health-care usage, and to assess whether this has led to a reduction in diagnoses, and therefore potentially missed or delayed diagnoses, of common physical and mental health conditions in this deprived urban population.

## Methods

### Study design and data sources

We did a retrospective cohort study, using anonymised electronic health records obtained from the Salford Integrated Record database between Jan 1, 2010, and May 31, 2020. The database includes primary care data collected from 47 general practices in Salford and is automatically updated nightly.^12^ The Salford Integrated Record governance board granted approval for the proposal and all patient data was de-identified, thus the need for ethical approval and patient consent was waived.

### Procedures

To determine the potential impact of the COVID-19 emergency on missed diagnoses, we used the number of first diagnoses for four disease groups: common mental health problems, cardiovascular and cerebrovascular disease, type 2 diabetes, and cancer (table 1). First diagnosis was used to avoid scenarios in which diagnosis codes were re-entered during clinical reviews, indicating that the same diagnosis has been made more than once, when only a single episode had occurred.

**Table 1:** Specific illnesses included within each of the four broad diagnostic groups assessed

	**Conditions included**	**Medication**
Circulatory system diseases	Atrial fibrillation, coronary heart disease (including myocardial infarction), deep vein thrombosis, heart failure, hypertension, peripheral arterial disease, pulmonary embolism, stroke, transient ischaemic attack	Clopidogrel, angiotensin-converting enzyme inhibitors, dihydropyridine calcium channel blockers, aspirin 75 mg
Type 2 diabetes	Type 2 diabetes	Metformin
Common mental health problems	Anxiety disorders, depression	Selective serotonin reuptake inhibitors
Malignant cancer	Bladder cancer, breast cancer, brain or CNS cancer, cervical cancer, colorectal cancer, kidney cancer, leukaemia, liver cancer, lung cancer, melanoma, myeloma, non-Hodgkin lymphoma, oesophageal cancer, oral cancer, ovarian cancer, pancreatic cancer, prostate cancer, stomach cancer, thyroid cancer	NA

NA=not applicable.

The Quality and Outcomes Framework, a financial incentivisation scheme, has been paused during the COVID-19 emergency.^13^ Payments are linked to the quality of care provided, as measured by their reporting via clinical codes. Primary care clinicians have also switched to remote working during the COVID-19 pandemic, which might have changed the way that they interact with their clinical information systems. For these reasons, and perhaps others, it is conceivable that any change in the rate of diagnosis is due to a change in recording behaviour, rather than an underlying change in the numbers of patients with new diagnoses. We therefore also extracted data for the issuing of new prescriptions of medications that are highly indicative of an underlying condition—eg, a prescription of metformin for a patient who has previously never been prescribed that drug is most likely due to a new diagnosis of type 2 diabetes (table 1). Malignant cancer is the only diagnosis group for which we did not extract prescription data, because the treatment of cancer in the UK is provided outside of primary care and therefore no prescription data would be available on patients' records to act as a proxy for a diagnosis. The recording of issued prescriptions (rather than collected prescriptions) in the UK is automated and therefore any temporal changes observed in prescribing patterns are likely to reflect actual fluctuations in incidence of newly diagnosed illnesses rather than changes in reporting. The dataset consisted of Read v2 codes and the clinical code sets^14^ for the diagnoses and medications were created systematically using our term set methodology, which has been described previously,^15^ and reviewed by two primary care clinicians (BB and OT).

### Data analysis

We extracted weekly numbers of clinical codes entered into patient records between Jan 1, 2010, and May 31, 2020, classified into the following high-level categories: symptoms and observations; diagnoses; prescriptions; operations and procedures; laboratory tests; and other diagnostic procedures. We plotted counts for the total weekly number of recorded clinical codes, overall and for each high-level category.

To investigate potential missed diagnoses, we extracted monthly counts of first diagnoses and first prescriptions for the conditions and medications of interest entered into patient records between Jan 1, 2010, and May 31, 2020. Negative binomial regression models were fitted on the monthly counts for the period Jan 1, 2010, to Feb 29, 2020. Due to low weekly numbers for some conditions and medications of interest, all data were formatted as monthly time-series data. Patients were the unit of observation, and month was the unit of analysis. The monthly counts were used as the outcome in the models and, to account for possible seasonality and potential time slopes, month was fitted as a categorical fixed-effect parameter and time as a continuous parameter. Each model was then used to predict the expected number of first diagnoses and first prescriptions between March 1 and May 31, 2020, and to calculate accompanying 95% CIs of these values, for each condition and prescribed medication type. The expected values were then compared and plotted against the observed counts in the dataset; if the observed values fell outside of the 95% CI range, then the difference between observed and expected values was considered significant. The CI calculated was the 95% prediction interval.^16^ The prediction interval accounts for uncertainty in estimating the population mean, and random variation of individual values. Future values predicted from the model would be expected to fall within the calculated prediction interval 95% of the time. The observed values were considered fixed with no sampling variability.

This study adhered to the REporting of studies Conducted using Observational Routinely-collected Data reporting guidelines^17^ (appendix pp 18–22). All statistical analyses were done using R (version 4.0.0).

### Role of the funding source

The funder of the study had no role in study design, data collection, data analysis, data interpretation, or writing of the report. The corresponding author had full access to all of the data and the final responsibility to submit for publication.

## Results

Our dataset included 241 458 active individuals, of whom 119 394 (49%) were women. The mean age of patients within the dataset was 35 years (IQR 21–54). Of the 200 530 individuals included in the dataset who had a known postcode, 93 257 (47%) resided in postcode areas in the most deprived quintile nationally and 17 832 (9%) lived in areas in the least deprived quintile nationally according to The English Indices of Deprivation.^9^

**Figure 1:**
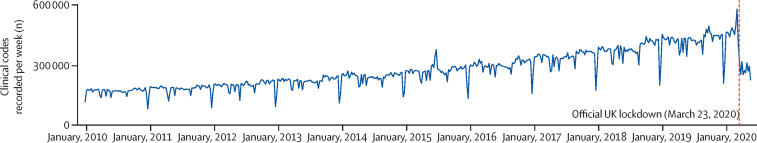
Number of clinical codes recorded in patient records per week, 2010–20 Clinical codes included all symptoms, observations, diagnoses, prescriptions, operations, procedures, laboratory tests, and administration codes.

In the past 10 years, the recording of all types of clinical code has increased. However, a large reduction in recording of clinical codes was observed after the onset of the COVID-19 emergency (figure 1). The reduction in activity was similar to that typically observed each year during the Christmas period, but was sustained since just before the emergency began in the middle of March through to May 31, 2020, when the observation period ended.

**Figure 2:**
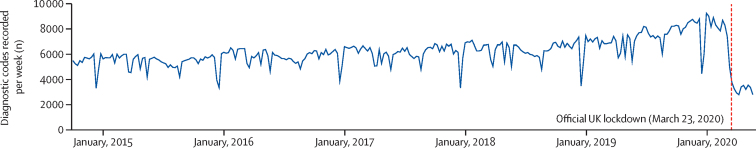
Number of diagnostic codes recorded in patient records per week, 2015–20 Counts included all diagnostic codes in patient records, not limited to the four diagnostic categories assessed in this study.

**Figure 3:**
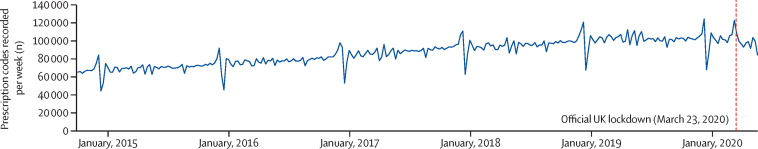
Number of prescription codes recorded in patient records per week, 2015–20 Counts include all prescription codes in patient records, not limited to the individual prescriptions assessed in this study.

A reduction in the number of clinical codes reported per week was observed for all categories of clinical code assessed, including diagnostic codes (figure 2), with the exception of medication prescriptions. The number of clinical codes reported for medication prescriptions increased before lockdown, followed by a more modest reduction in reporting after March 23, 2020, compared with that observed for the other categories (figure 3). Time-series charts for the other categories of clinical code are included in the appendix (pp 1–3).

The observed number of first diagnoses of each of the four disease groups between March and May, 2020, decreased significantly when compared with the expected numbers. As estimated from the regression model, 2147 diagnoses (95% CI 1821 to 2489) of common mental health problems were expected between March 1 and May 31, 2020. In the same period, 1073 diagnoses were recorded, representing 1074 (95% CI 748 to 1416) fewer diagnoses of common mental health problems, and a 50·0% decline (95% CI 41·1 to 56·9) compared with previous years (table 2). 456 fewer diagnoses of circulatory system diseases were recorded (43·3% reduction, 95% CI 29·6 to 53·5) and 135 fewer diagnoses of type 2 diabetes (49·0% reduction, 23·8 to 63·1). 31 fewer cancer diagnoses were recorded, but this reduction was not statistically significant (16·0% reduction, 95% CI −18·1 to 36·6). However, the reduction in cancer diagnoses observed in May, 2020 was significant (44·1% reduction, 95% CI 22·4 to 57·8; appendix p 16).

**Table 2:** Difference between the expected and observed number of first diagnoses or first prescriptions between March 1 and May 31, 2020

	**Expected cases, n (95% CI)**	**Observed cases, n**	Percentage reduction in cases, % (95% CI)*
Type 2 diabetes	276 (185 to 382)	141	49·0% (23·8 to 63·1)
Metformin	331 (248 to 422)	213	35·7% (14·1 to 49·5)
Circulatory system disease	1054 (850 to 1286)	598	43·3% (29·6 to 53·5)
Aspirin 75 mg	301 (228 to 381)	213	29·3% (6·6 to 44·1)
Calcium channel blockers	558 (452 to 668)	359	35·6% (20·6 to 46·3)
ACEIs	518 (414 to 632)	249	52·0% (39·9 to 60·6)
Clopidogrel	265 (179 to 367)	148	44·2% (17·3 to 59·7)
Common mental health problems	2147 (1821 to 2489)	1073	50·0% (41·1 to 56·9)
SSRIs	737 (593 to 889)	449	39·1% (24·3 to 49·5)
Malignant cancer	194 (138 to 257)	163	16·0% (−18·1 to 36·6)

Expected numbers of first diagnoses and prescriptions were generated using negative binomial regression models. ACEIs=angiotensin-converting enzyme inhibitors. SSRIs=selective serotonin reuptake inhibitors.

* Expected cases minus observed cases.

The observed number of first prescriptions of medicines commonly used to treat the four disease groups (common mental health problems, cardiovascular and cerebrovascular disease, type 2 diabetes, and cancer) was also lower than the expected number between March 1 and May 31, 2020. Compared with the expected numbers, 288 fewer first prescriptions of selective serotonin reuptake inhibitors were recorded (39·1% reduction, 95% CI 24·3 to 49·5), and 118 fewer first prescriptions of metformin (35·7% reduction, 14·1 to 49·5; table 2). The number of diagnoses and prescriptions recorded per month between March and May, 2020, and their expected values, are shown in the appendix (pp 15–16).

**Figure 4:**
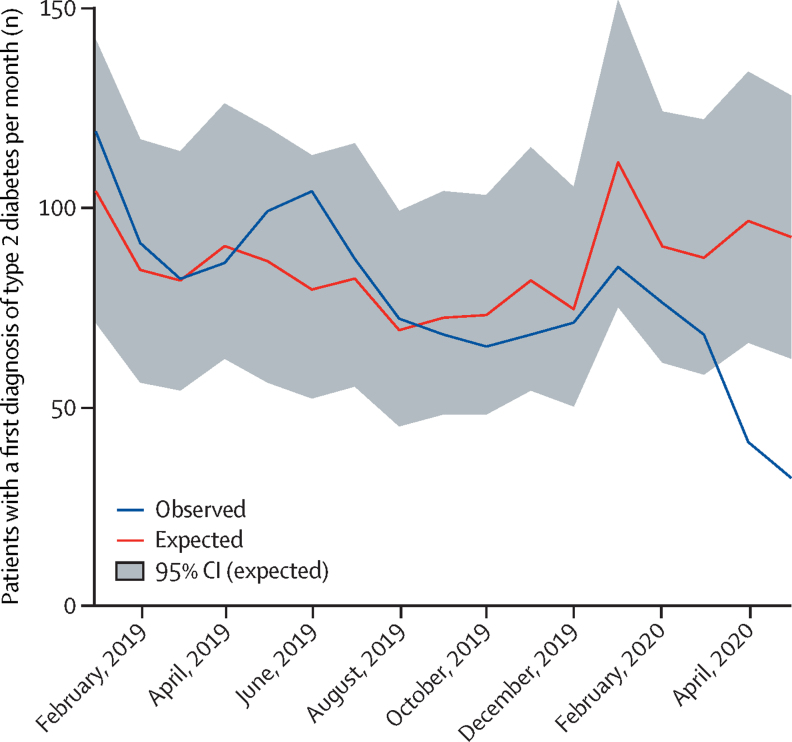
Temporal variation in the number of patients with a first diagnosis of type 2 diabetes per month, Jan 1, 2019–May 31, 2020 Number of expected cases (95% CI) was estimated with negative binomial regression models, using data from Jan 1, 2010, to Feb 29, 2020 (inclusive).

**Figure 5:**
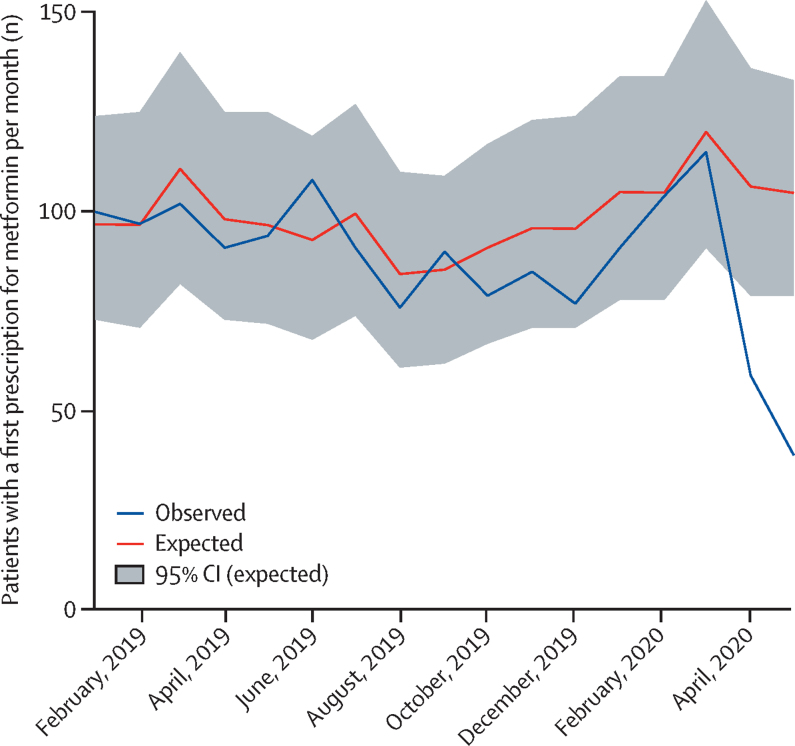
Temporal variation in the number of patients with a first prescription for metformin per month, Jan 1, 2019 –May 31, 2020 Number of expected cases (95% CI) was estimated with negative binomial regression models, using data from Jan 1, 2010, to Feb 29, 2020 (inclusive).

Between April and May, 2020, the observed number of patients with a first diagnosis of type 2 diabetes per week, and the associated first prescriptions of metformin per week were markedly lower than the numbers expected for that time period (Figure 4, Figure 5). A similar pattern was observed for the diagnoses of common mental health problems, cardiovascular and cerebrovascular disease, and cancer, and associated prescriptions (appendix pp 4–14).

We validated our analytical approach by developing negative binomial models based on data from Jan 1, 2010, to Feb 28, 2018. We then used the models to predict the expected values between March 1 and May 31, 2018, and confirmed that the observed values from the dataset were within the 95% prediction interval. We also repeated this exercise for 2019. For 2018 and 2019, the observed values for all diagnoses and prescriptions were within the 95% prediction interval (appendix p 17).

## Discussion

This population-based study of primary care electronic health records done in a deprived UK city has identified large reductions in the number of new diagnoses recorded for circulatory system diseases, type 2 diabetes, malignant cancers, and common mental health problems during the COVID-19 public health emergency. This pattern was mirrored by corresponding reductions in the number of new prescriptions for medications that are often used to treat these conditions. Universal automation of primary care prescription excludes the possibility that these diagnoses occurred but were not recorded. All UK patients are registered with a single GP who represents the first point of contact for new health problems. Therefore our dataset is likely to include the majority of primary health-care contacts for the population of this geographical region during the study period. Most of the conditions included in our study develop over many years, so it is unlikely that people's behaviour during the COVID-19 pandemic has resulted in a lower incidence of these diseases. With the exception of mental health, all of the diagnoses we assessed are determined by objective tests. Therefore, we do not believe that the steady increase observed over time in the number of patients diagnosed with these conditions is due to an increasing tendency to overdiagnose. Even for mental health problems, it is widely regarded that underdiagnosis rather than overdiagnosis remains a major problem, particularly in deprived populations, such as Salford. Therefore, the reduced number of new diagnoses observed when compared with the expected numbers obtained from our regression models, are most likely to represent a large number of true disease cases that have gone undetected, undiagnosed, and untreated.

To our knowledge, this is the first population-based study to assess and quantify the indirect impact of the COVID-19 pandemic on potential missed diagnoses in primary care. Strengths of our study include that the data assessed were from the entire population of one city, the study involved two local GPs (BB, OT) who would have been aware of any local intiatives that could have affected our results, and the study was developed by a multidisciplinary team.

In studies using routinely collected health-care data, it is often unclear whether a lower than expected frequency of diagnostic coding pertains to undiagnosed cases or underreporting of actual diagnoses. However, by corroborating reductions in first diagnoses with similar reductions in related prescribing, we have provided evidence that the observed reductions were most likely to be associated with missed diagnoses.

The data were obtained from a single urban area in England and thus might not be generalisable to other localities. This is particularly true considering the high levels of deprivation and premature mortality rates in Salford. A higher proportion of the population in Salford might be unable to consult with a GP via video link than the general population of the UK. Thus, the same reductions might not have been observed in other areas. However, it seems reasonable to assume that the patterns observed in Salford would approximate those in other parts of the UK, particularly in areas with similar populations. The high COVID-19 mortality rate in Salford should not affect the generalisability of our results, since we are reporting the consequences of the public health system response and the public response to the crisis rather than the consequences of COVID-19 itself. Additionally, focusing on the entire population of a single city enabled us to carefully scrutinise our data and interpretations against the clinical experiences of front-line staff and local policies, which would be harder to achieve when using larger, disparate, databases such as the Clinical Practice Research Datalink.^18^

Few studies have reported on the indirect impact of the COVID-19 emergency on missed diagnoses of common conditions that are associated with elevated mortality risk if not diagnosed promptly and effectively treated. Several publications have reported on the apparent impact of patients disengaging with health-care services, with reductions in emergency department attendance and emergency hospital admissions in the UK^3^, ^19^, ^20^ and in paediatric emergency department visits in Italy.^21^ Although our focus is primary care, several of the conditions that we assessed such as stroke or myocardial infarction, would typically present first in hospital, before long-term management in primary care. Therefore the 37% reduction in emergency admissions in the UK in April, 2020^22^ is consistent with the reductions observed in our study. Patients utilising other services such as general practice might explain the reduction in emergency admissions.^19^ However, the reductions observed for reported clinical codes, and the reductions in first diagnoses and prescriptions in primary care observed in this study, are not consistent with this theory; patients seem to be avoiding all clinical settings rather than using alternatives.

A survey of the members of the Royal College of Psychiatrists in the UK found that of 1369 respondents, 43% reported an increase in urgent or emergency cases of mental health care, compared with 22% who reported a decrease. However non-urgent clinical activity (ie, routine appointments and interventions) has decreased, with 45% of respondents reporting a decrease in routine appointments, and only 9% reporting an increase.^22^ Combined with the high levels of reported anxiety (49·6% of people in the UK reported high anxiety in late March, 2020), this unmet need could result in a surge in demand in the coming months.^23^, ^24^

For malignant cancers, a backlog of patients with potential symptoms in the UK is expected following the COVID-19 emergency.^25^ Furthermore, a 34·3% reduction in the number of urgent referrals for diagnosis in the UK was reported between February and April, 2020,^7^ with Lai and colleagues reporting a reduction of 70–89% for the same time period.^8^ We observed a modest, non-significant, reduction (16·0%) in the number of malignant cancer diagnoses between March 1 and May 31, 2020. We hypothesise that the lack of a significant difference might be because only the most serious cases, and therefore individuals most likely to receive a positive diagnosis, are presenting. In a report from the Salford Royal NHS Foundation Trust, a reduction of 53% in skin cancer diagnoses was predicted between March and April, 2020.^7^ There is a delay between the time when a patient receives a cancer diagnosis in secondary care, and the data being entered into their primary care record.^26^ Therefore, the lower reduction in cancer diagnoses observed compared with the other diseases assessed could be partially explained by this time lag. This hypothesis is supported by the data for May, during which we observed a 44·1% reduction (95% CI 22·4–57·8) in cancer diagnoses, which was statistically significant.

For circulatory system diseases, existing evidence suggests that many patients avoided health-care settings during the COVID-19 emergency, with a 70% reduction in patients presenting with myocardial infarction in Lombardy (Italy), a 40% reduction across Spain, and a 20–50% reduction in Atlanta (GA, USA).^27^ Lifestyle and environmental changes might have affected the true incidence in the community, but evidence from Hong Kong suggests that instead patients with these conditions presented with symptoms at a more advanced stage than would be expected before the COVID-19 pandemic.^28^

Major negative impacts are not inevitable as a consequence of a decrease in primary care utilisation. For minor illnesses, people might seek alternative solutions, or the problem might be resolved without medical intervention. However, this is not true for the conditions assessed in this study, which will mostly not resolve without intervention. The conditions might be long term and progressive, especially when not treated. Diagnostic delays for these conditions have been associated with increased mortality and poorer outcomes in patients with myocardial infarction^29^ and depression.^30^

When frequency of engagement with health services increases again, through less widespread fear of contracting COVID-19 in a health-care facility or because patients' symptoms have become intolerable, presentation rates for the four groups of conditions assessed might markedly increase. Should such a scenario transpire, health-care services will need to manage this excess demand. The delay in diagnoses is also likely to have implications for the severity of these conditions when patients present. Prioritisation of people with these conditions over people with more minor illnesses will be important in primary care otherwise the backlog could plausibly overwhelm primary and secondary health-care services. New diagnoses of conditions that have increased due to the pandemic will pose additional challenges.^24^, ^25^, ^27^ If a similar emergency occurs in the future, steps should be taken to mitigate these indirect effects. One possible option would be to carefully construct public communications to ensure that patients continue to use health-care services appropriately as and when needed. Another option is to ensure that remote consultations, by telephone or video chat, remain widespread and normalised, so that patients can continue to engage with health services even when they have concerns regarding the potential consequences of physically attending a health-care setting.

Our study shows that the COVID-19 pandemic has resulted in a large number of potentially missed or delayed diagnoses of health conditions, which carry high risk if not promptly diagnosed and effectively treated. Primary and secondary care services must proactively prepare to address the large backlog of patients that is likely to follow. Should a public health emergency on the scale of the COVID-19 pandemic occur in the future, or if subsequent surges in COVID-19 cases arise, national communication strategies must be carefully considered to ensure that large numbers of patients with urgent health needs do not disengage with health services.

## Data sharing

All data, code, and clinical code sets are publicly available.
