# Supplementary material for: Diagnosis of physical and mental health conditions in primary care during the COVID-19 pandemic: a retrospective cohort study
Source: Lancet Public Health. 2020 Sep 23;5(10):e543–50. doi: 10.1016/S2468-2667(20)30201-2 (PMC7511209; doi:10.1016/S2468-2667(20)30201-2)
Supplement: Supplementary appendix [file mmc1.pdf]

THE LANCET
Public Health

Supplementary appendix

This appendix formed part of the original submission and has been peer reviewed. We post it as supplied by the authors.

Supplement to: Williams R, Jenkins DA, AshcroftDM, et al. Diagnosis of physical and mental health conditions in primary care during the COVID-19 pandemic: a retrospective cohort study. *Lancet Public Health* 2019; published online Sept 23. https://doi.org/10.1016/S2468-2667(20)30201-2.

Supplementary material

Timeseries charts

Timeseries charts for each type of clinical code.

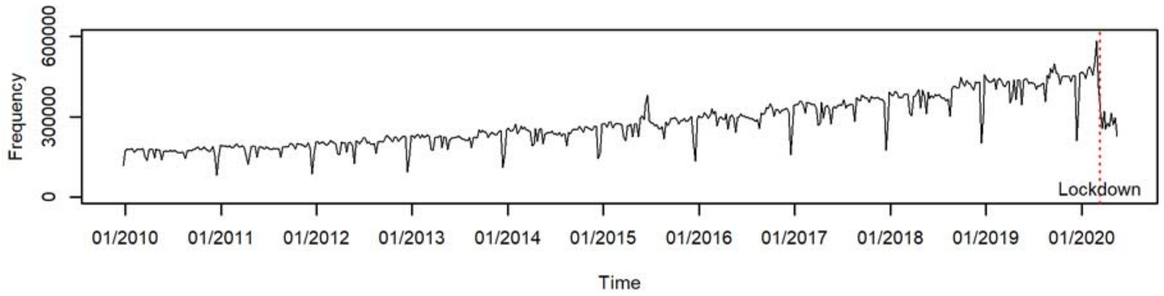

Figure S1 - Number of clinical codes recorded in patients' records each week since 2010. UK official lockdown (23rd March 2020) marked by red vertical line.

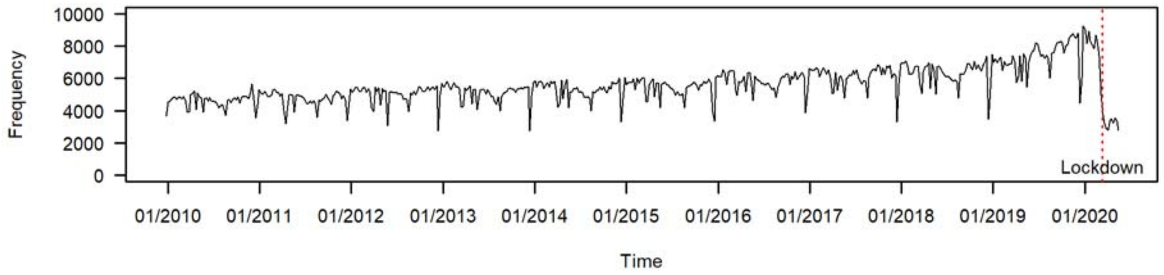

Figure S2 - Number of diagnosis codes recorded in patients' records each week since 2010. UK official lockdown (23rd March 2020) marked by red vertical line.

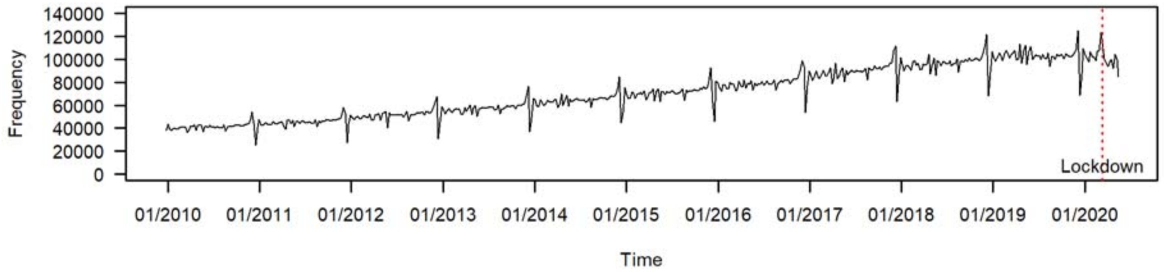

Figure S3 - Number of prescription codes recorded in patients' records each week since 2010. UK official lockdown (23rd March 2020) marked by red vertical line.

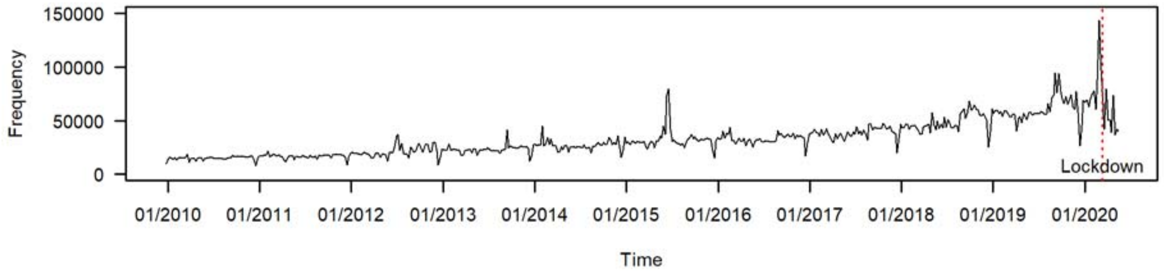

Figure S4 - Number of administration codes recorded in patients' records each week since 2010. UK official lockdown (23rd March 2020) marked by red vertical line. The large spike at lockdown is due to the recording of contact telephone numbers in order to facilitate remote consultations and contact via text message.

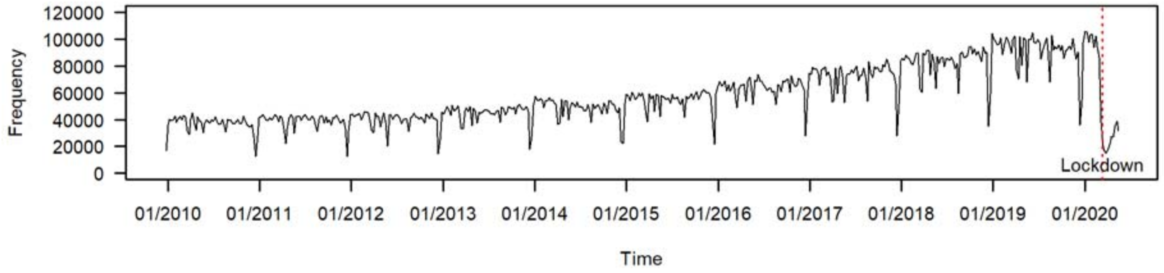

Figure S5 - Number of laboratory test result codes recorded in patients' records each week since 2010. UK official lockdown (23rd March 2020) marked by red vertical line.

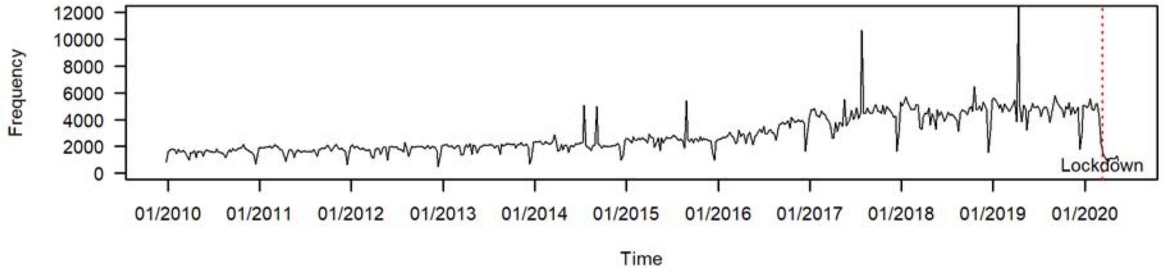

Figure S6 - Number of diagnostic procedure codes recorded in patients' records each week since 2010. UK official lockdown (23rd March 2020) marked by red vertical line. The spikes in mid-2017 and mid-2019 are when an electronic health record (EHR) vendor, bulk updated patients' records with a frailty score.

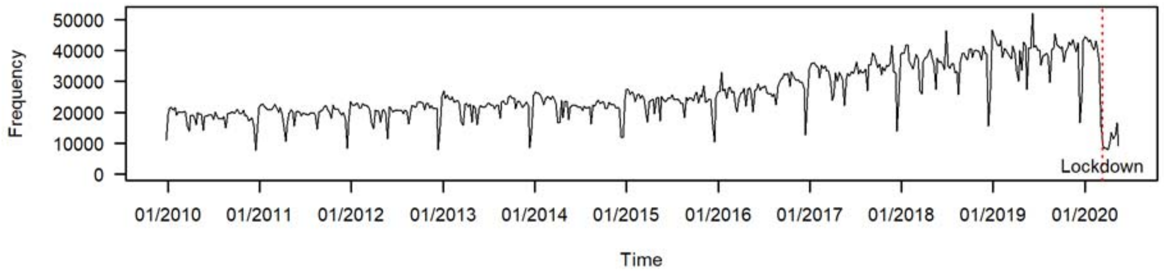

Figure S7 - Number of observation/symptom codes recorded in patients' records each week since 2010. UK official lockdown (23rd March 2020) marked by red vertical line.

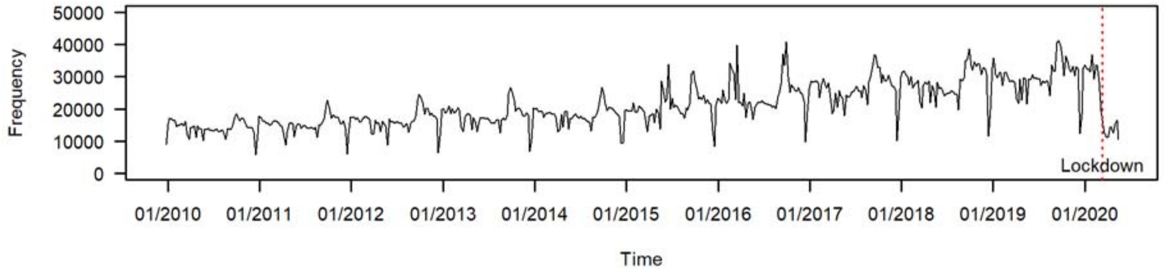

Figure S8 - Number of other procedure codes recorded in patients' records each week since 2010. UK official lockdown (23rd March 2020) marked by red vertical line.

Negative binomial regression charts

Charts showing the observed number of first diagnoses or first prescriptions for each condition and medication analysed.

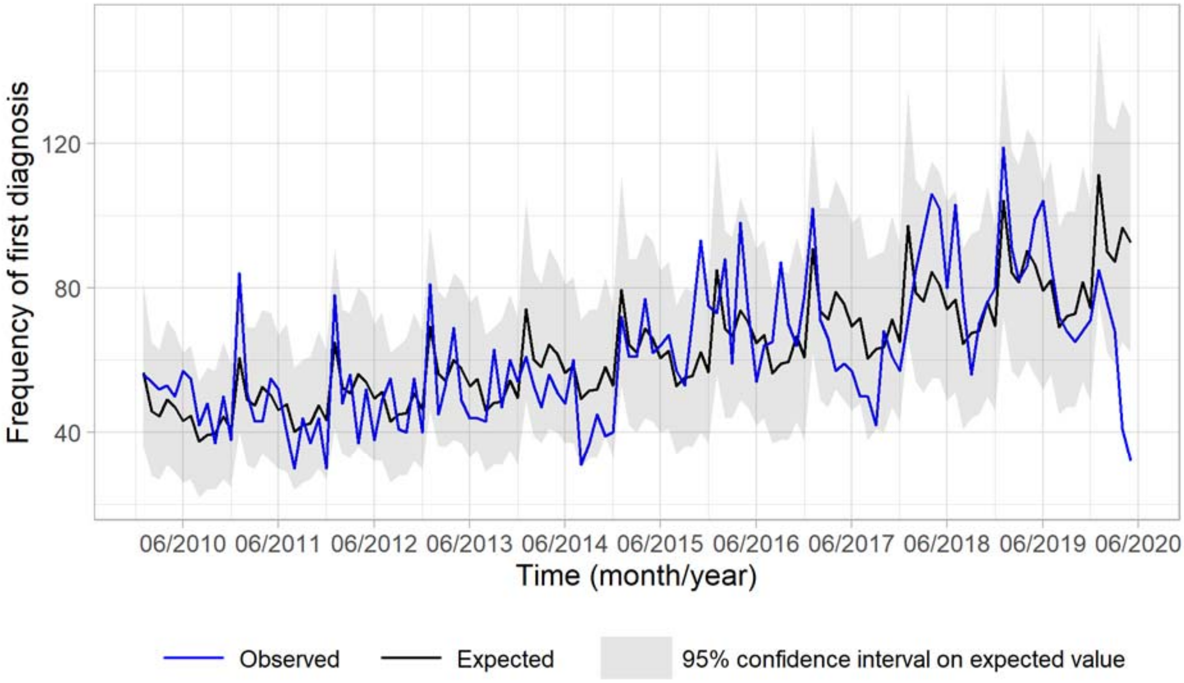

Figure S9 - First diagnosis of type 2 diabetes each month 2010-present. Expected line based on negative binomial regression model using data up to and including Feb 2020

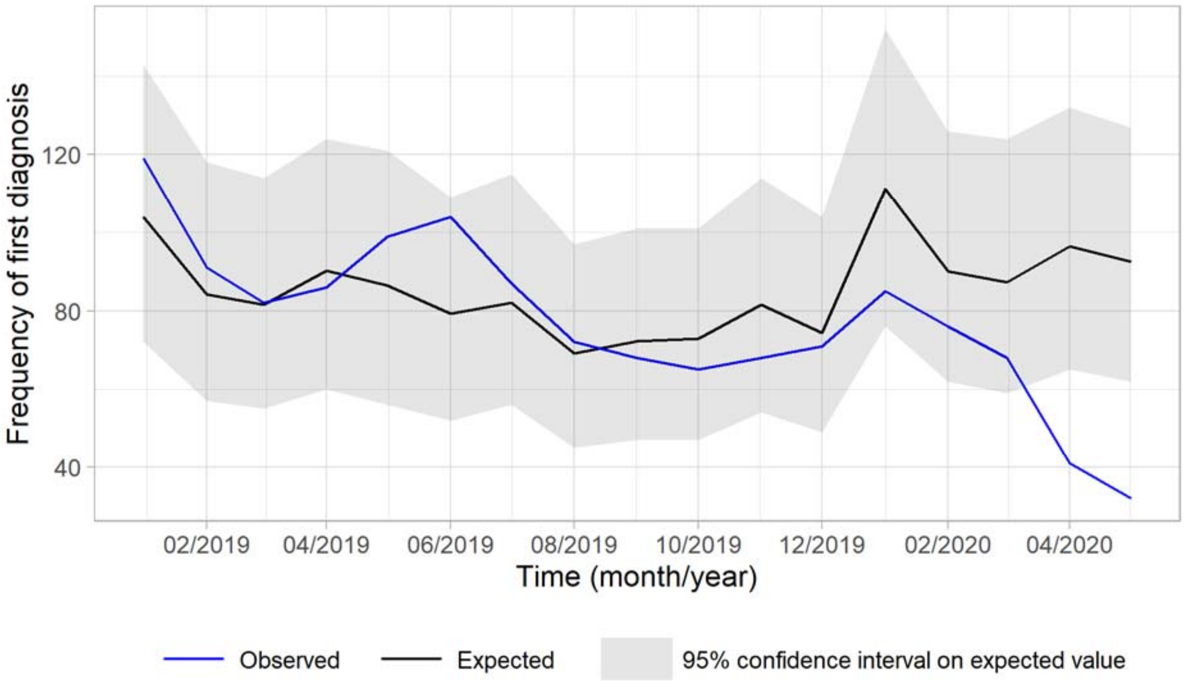

Figure S10 - First diagnosis of type 2 diabetes each month 2019-present. Expected line based on negative binomial regression model using data up to and including Feb 2020

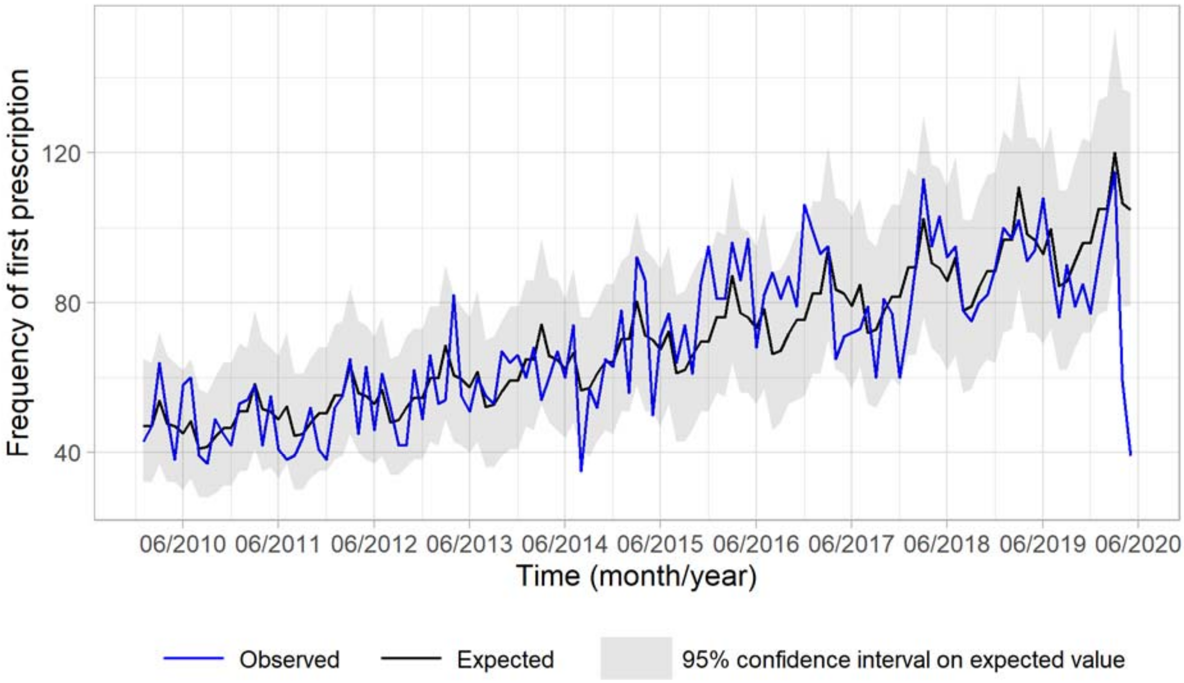

Figure S11 - First prescription of metformin each month 2010-present. Expected line based on negative binomial regression model using data up to and including Feb 2020

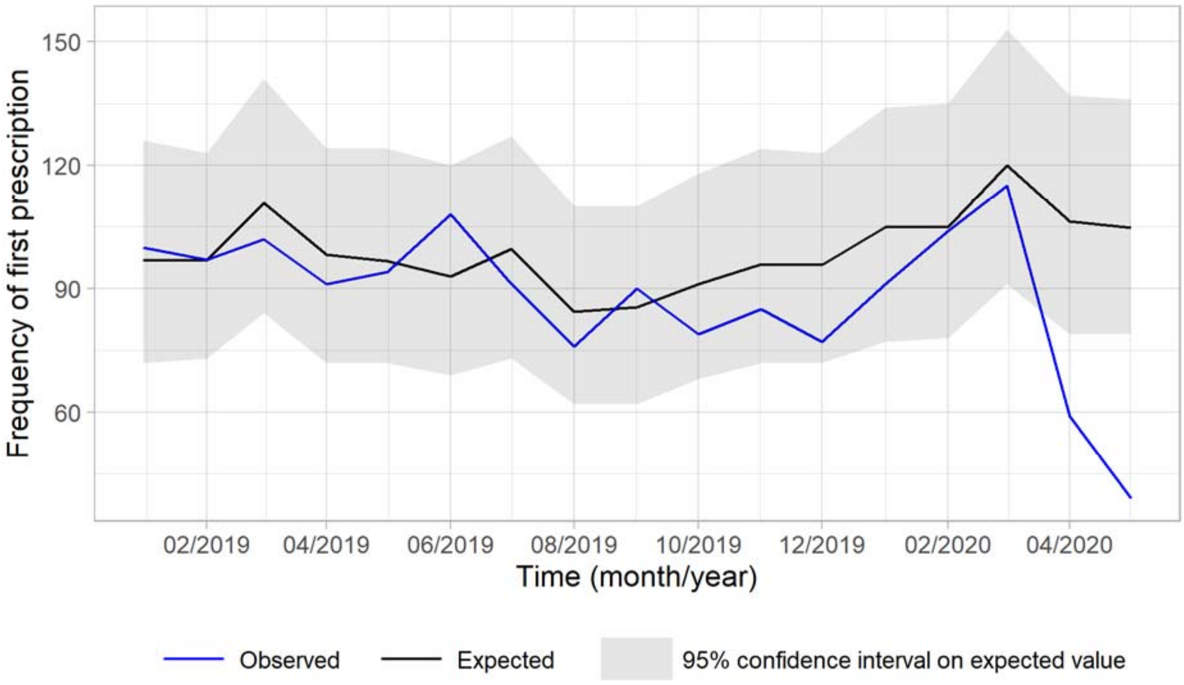

Figure S12 - First prescription of metformin each month 2019-present. Expected line based on negative binomial regression model using data up to and including Feb 2020

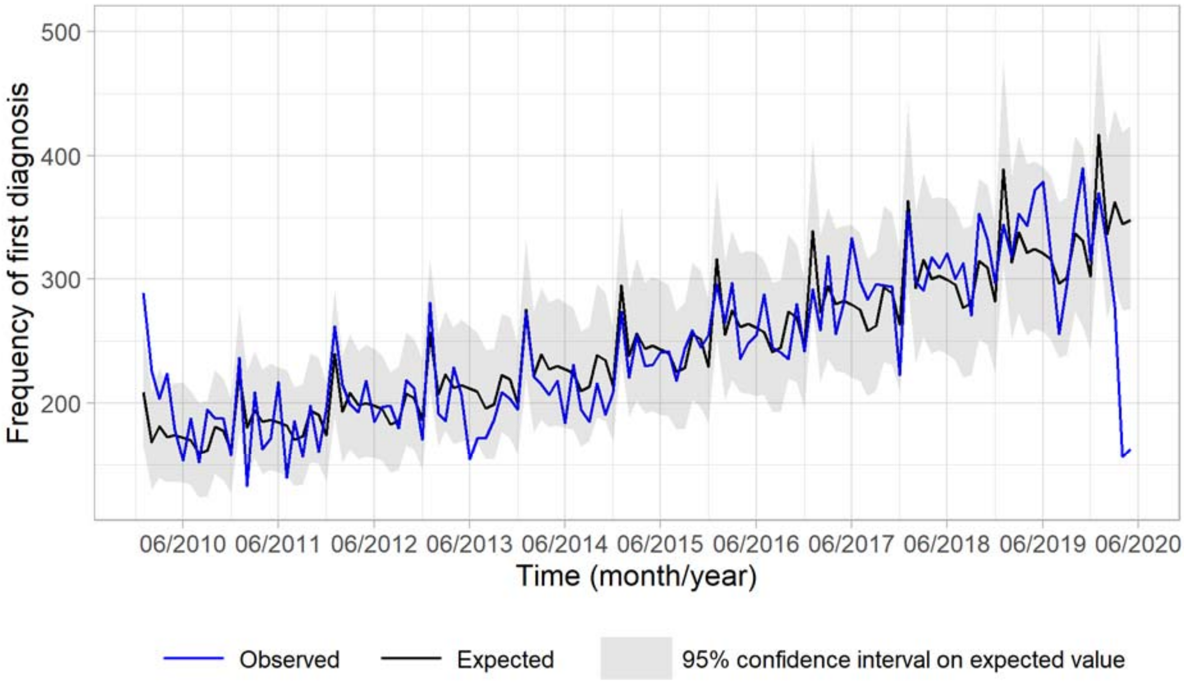

Figure S13 - First diagnosis of circulatory system disease each month 2010-present. Expected line based on negative binomial regression model using data up to and including Feb 2020

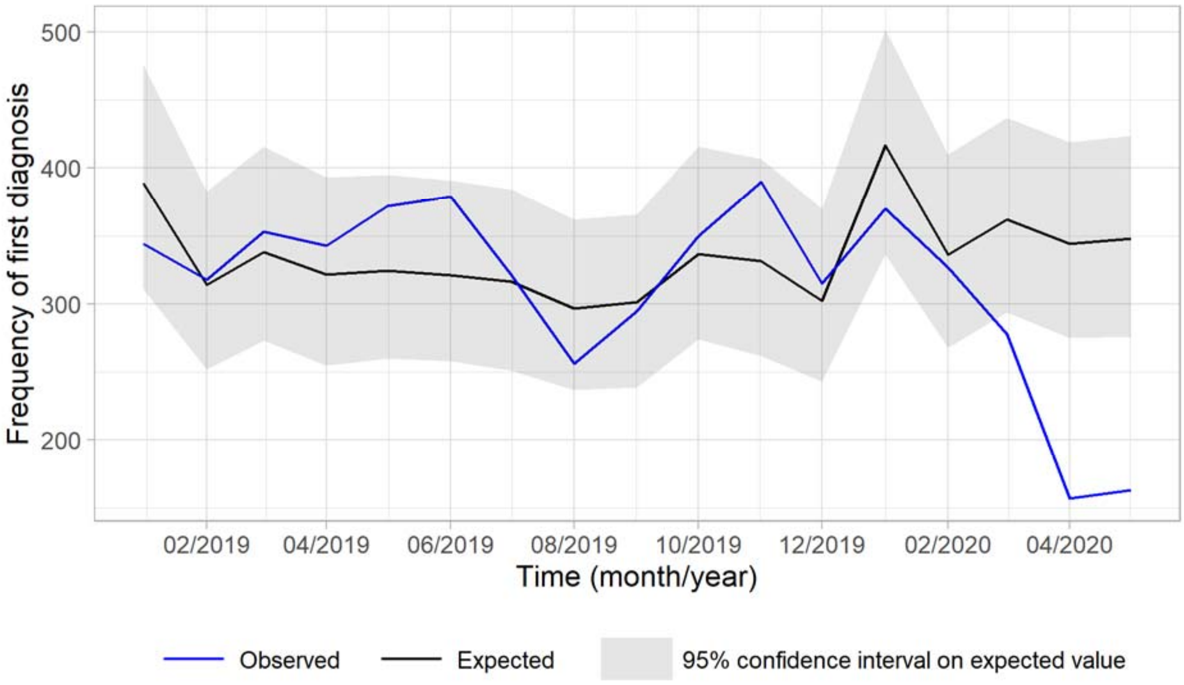

Figure S14 - First diagnosis of circulatory system disease each month 2019-present. Expected line based on negative binomial regression model using data up to and including Feb 2020

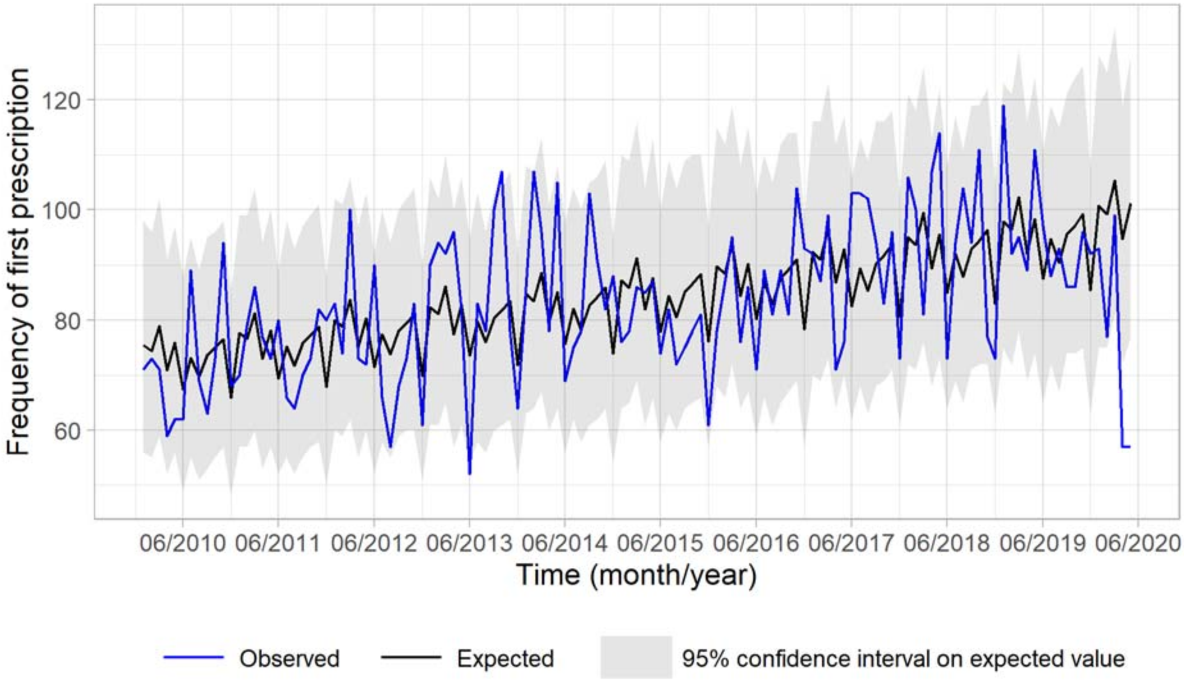

Figure S15 - First prescription of aspirin 75mg each month 2010-present. Expected line based on negative binomial regression model using data up to and including Feb 2020

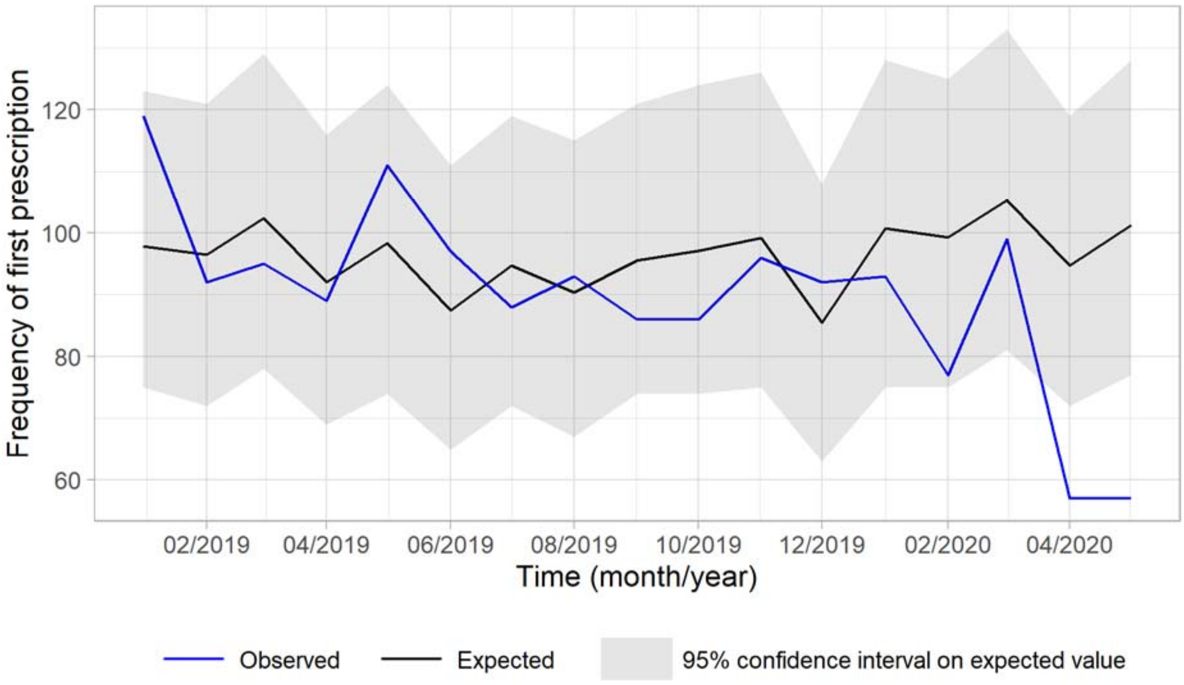

Figure S16 - First prescription of aspirin 75mg each month 2019-present. Expected line based on negative binomial regression model using data up to and including Feb 2020

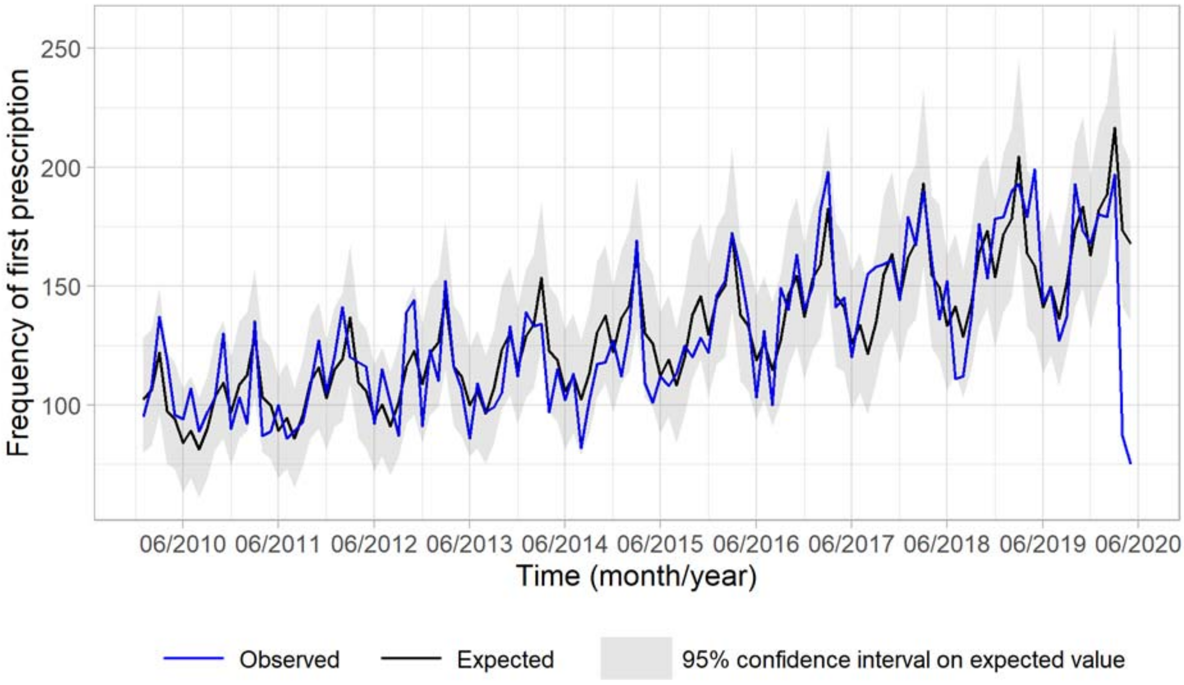

Figure S17 - First prescription of a dihydropyridine calcium channel blocker (CCB) each month 2010-present. Expected line based on negative binomial regression model using data up to and including Feb 2020

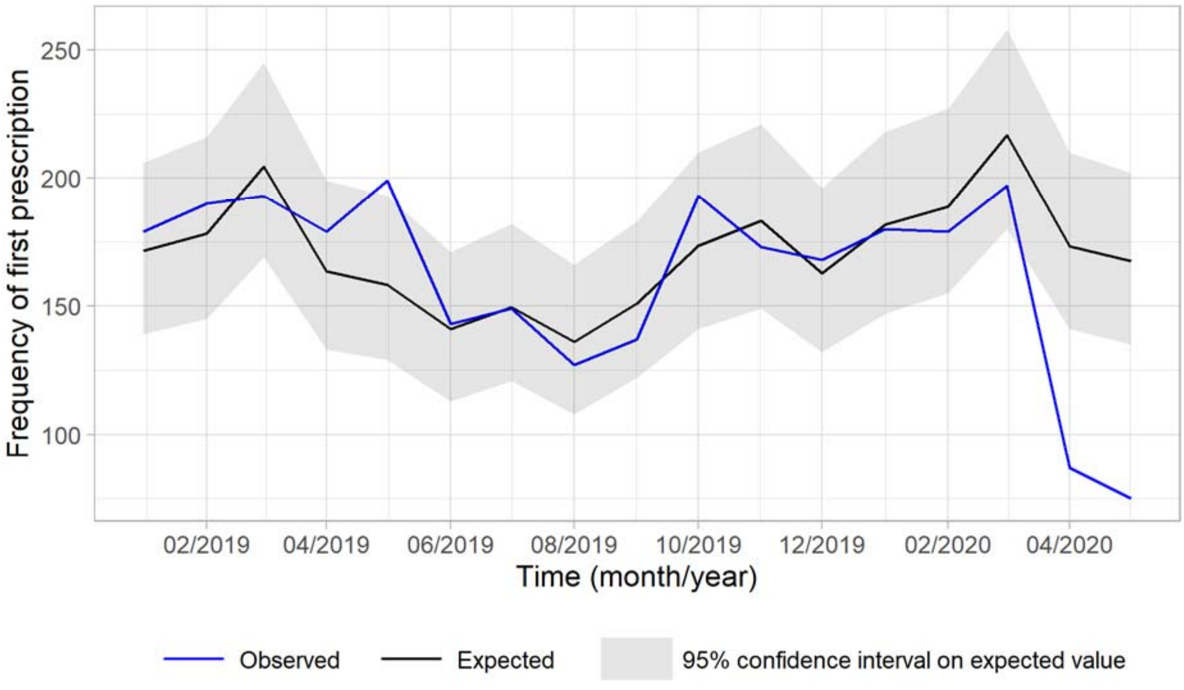

Figure S18 - First prescription of dihydropyridine calcium channel blocker (CCB) each month 2019-present. Expected line based on negative binomial regression model using data up to and including Feb 2020

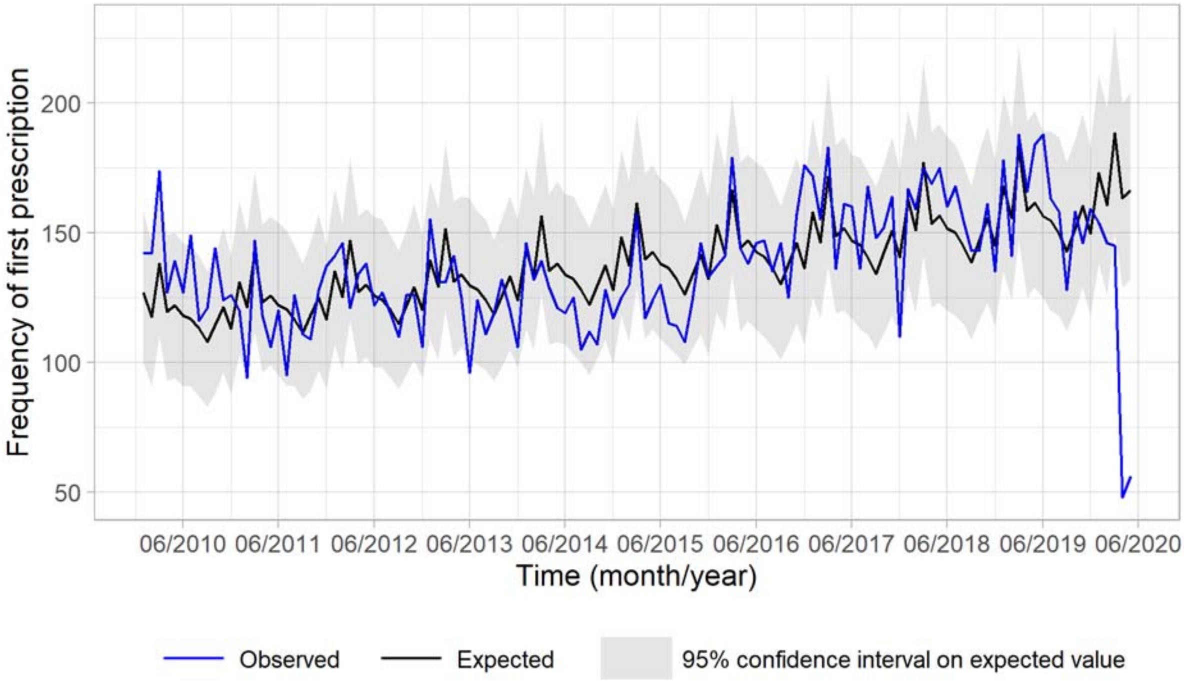

Figure S19 - First prescription of an angiotensin-converting enzyme inhibitor (ACEI) each month 2010-present. Expected line based on negative binomial regression model using data up to and including Feb 2020

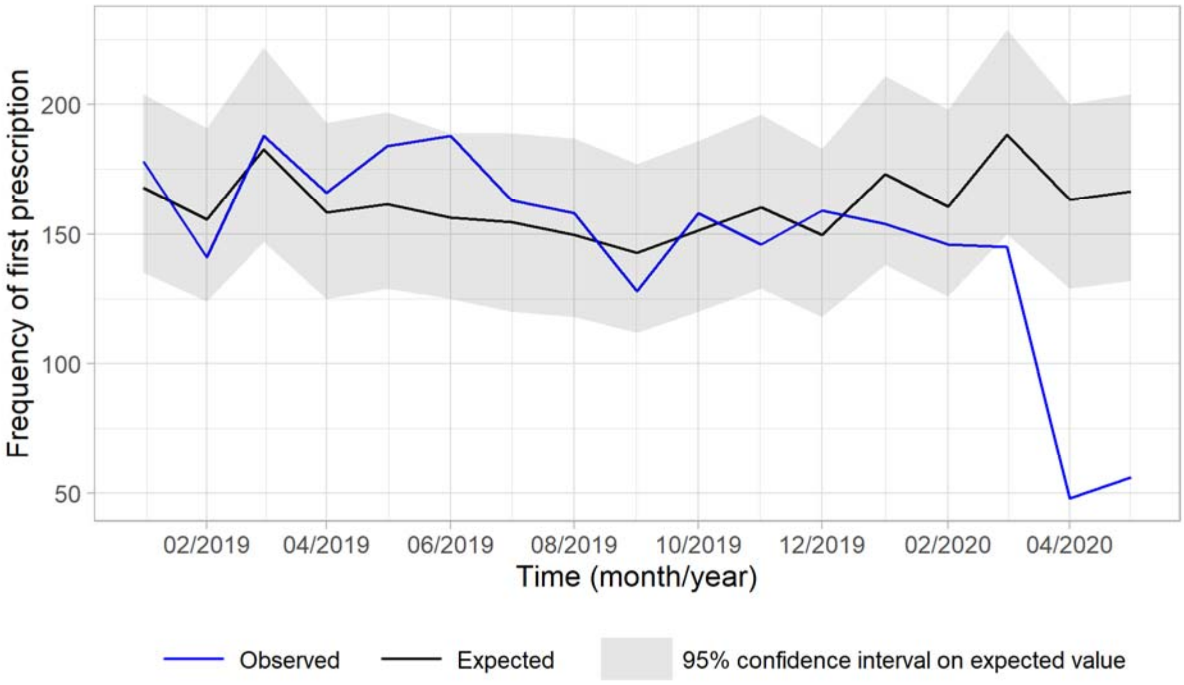

Figure S20 - First prescription of an angiotensin-converting enzyme inhibitor (ACEI) each month 2019-present. Expected line based on negative binomial regression model using data up to and including Feb 2020

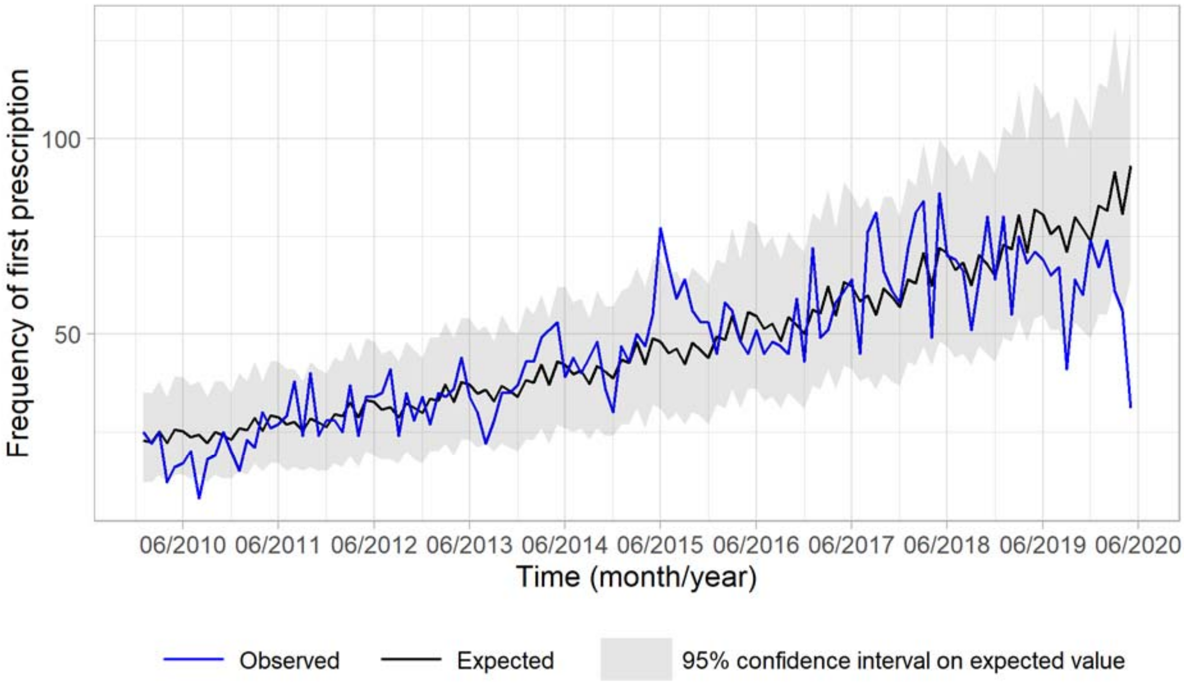

Figure S21 - First prescription of clopidogrel each month 2010-present. Expected line based on negative binomial regression model using data up to and including Feb 2020

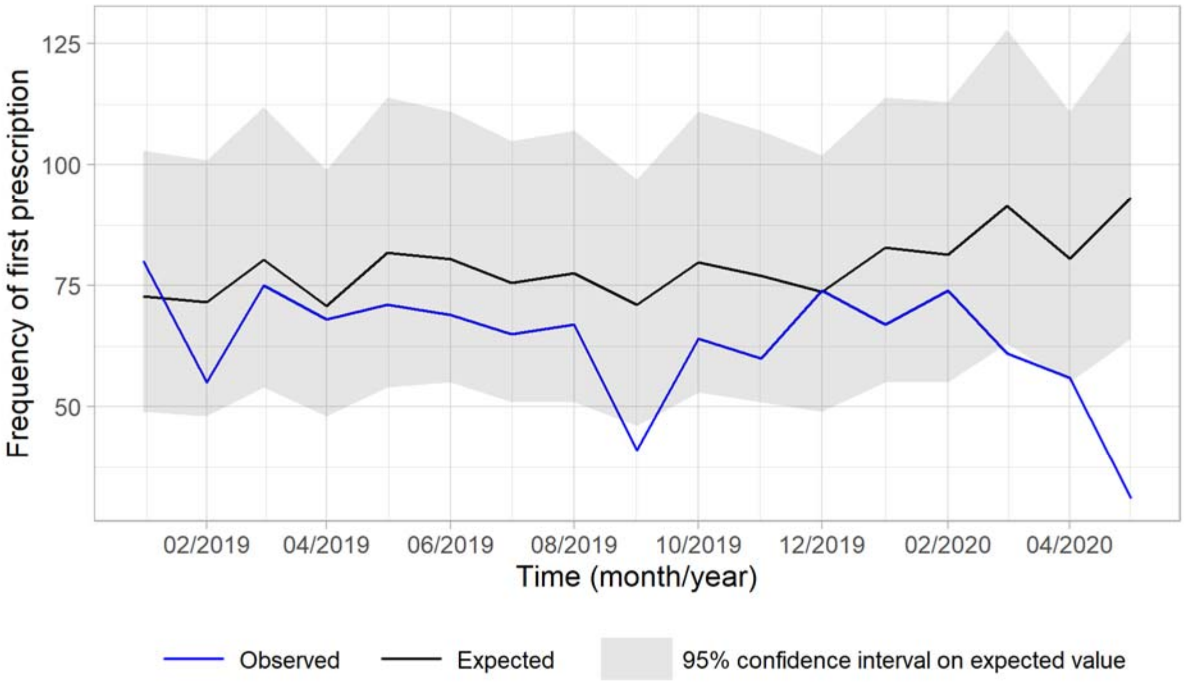

Figure S22 - First prescription of clopidogrel each month 2019-present. Expected line based on negative binomial regression model using data up to and including Feb 2020

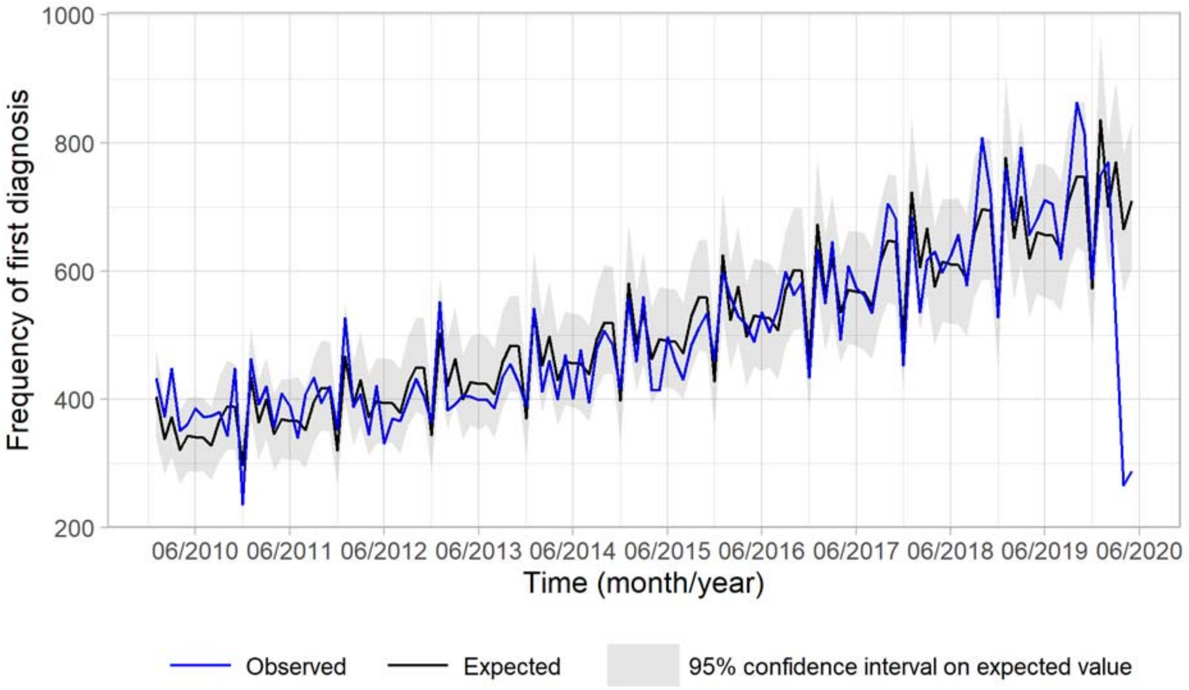

Figure S23 - First diagnosis of a common mental health problem each month 2010-present. Expected line based on negative binomial regression model using data up to and including Feb 2020

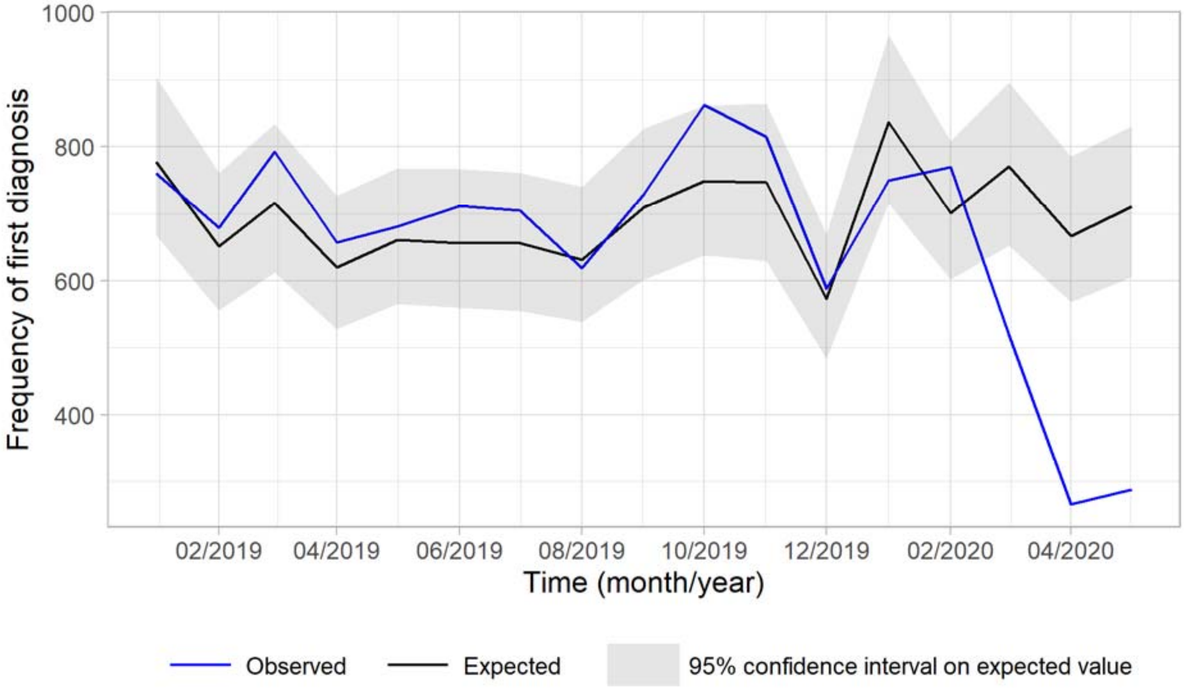

Figure S24 - First diagnosis of a common mental health problem each month 2019-present. Expected line based on negative binomial regression model using data up to and including Feb 2020

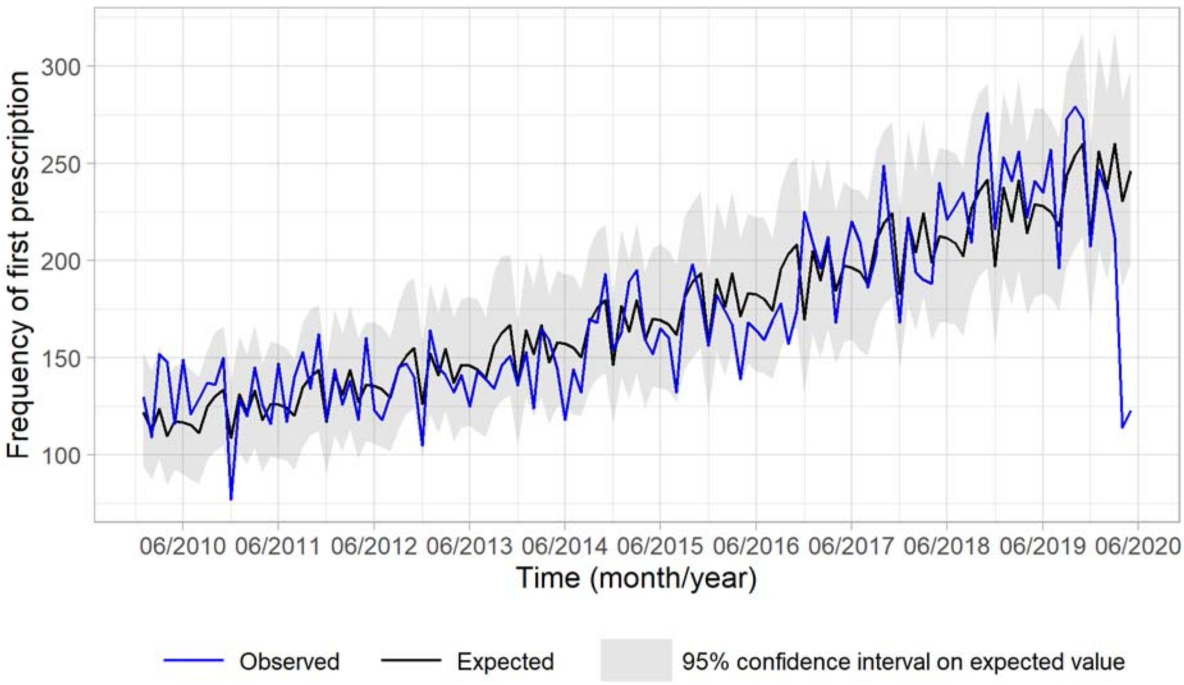

Figure S25 - First prescription of a selective serotonin reuptake inhibitor (SSRI) each month 2010-present. Expected line based on negative binomial regression model using data up to and including Feb 2020

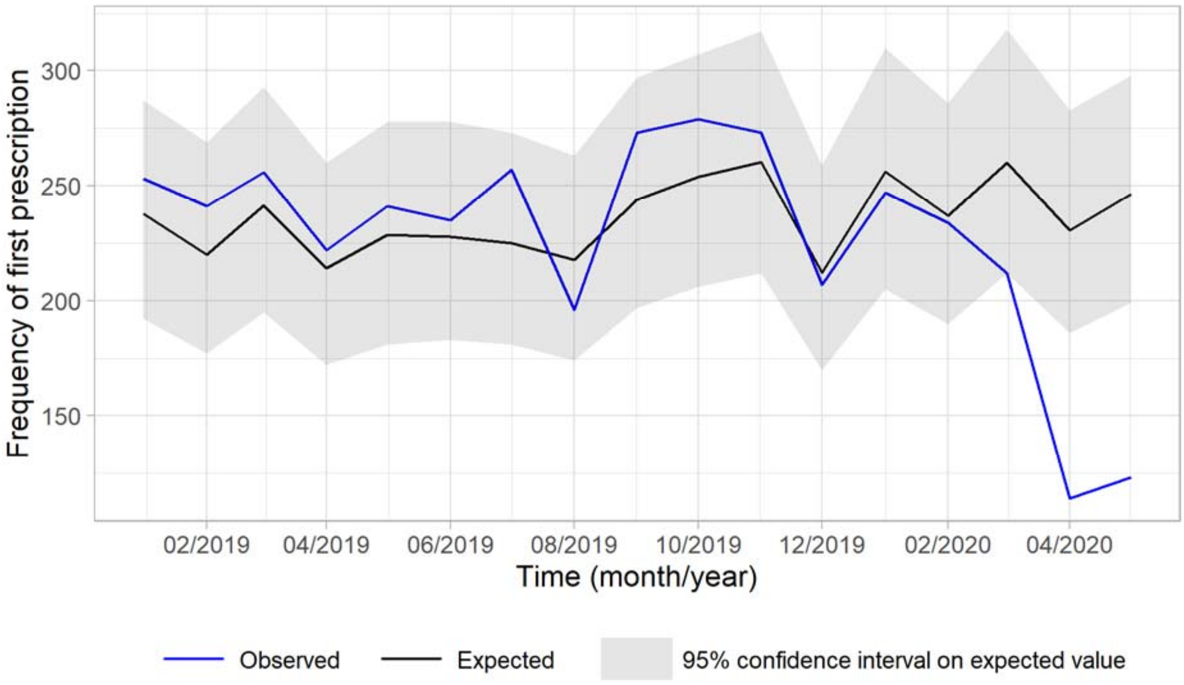

Figure S26 - First prescription of selective serotonin reuptake inhibitor (SSRI) each month 2019-present. Expected line based on negative binomial regression model using data up to and including Feb 2020

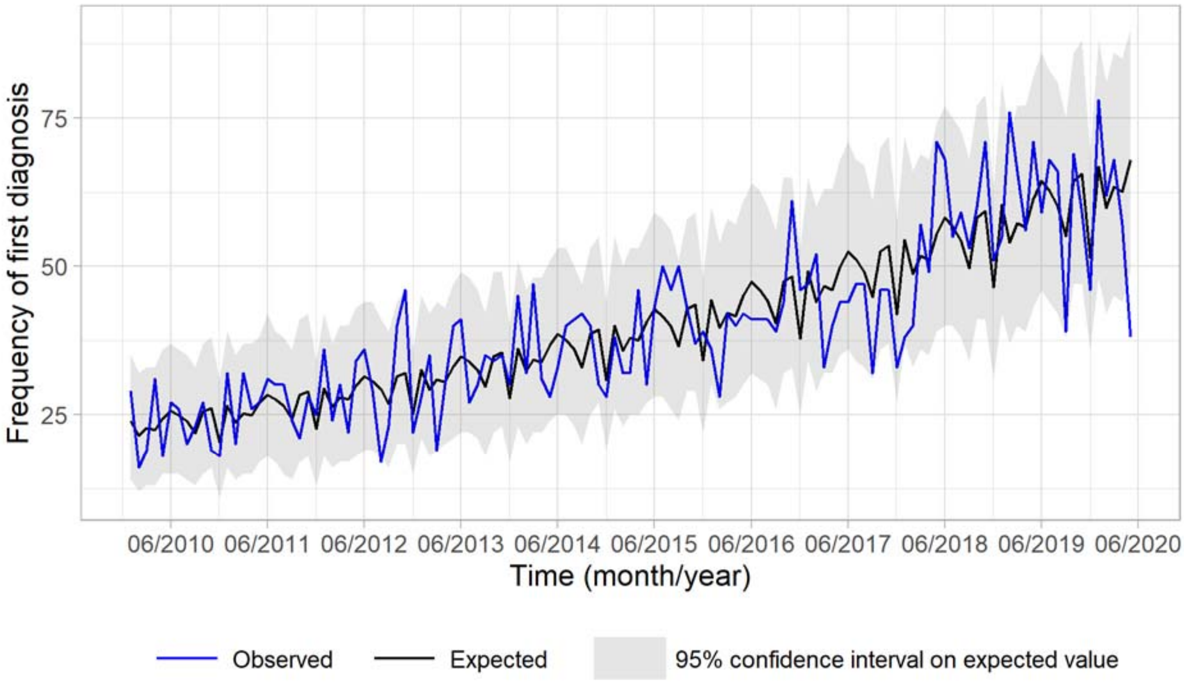

Figure S27 - First diagnosis of malignant cancer each month 2010-present. Expected line based on negative binomial regression model using data up to and including Feb 2020

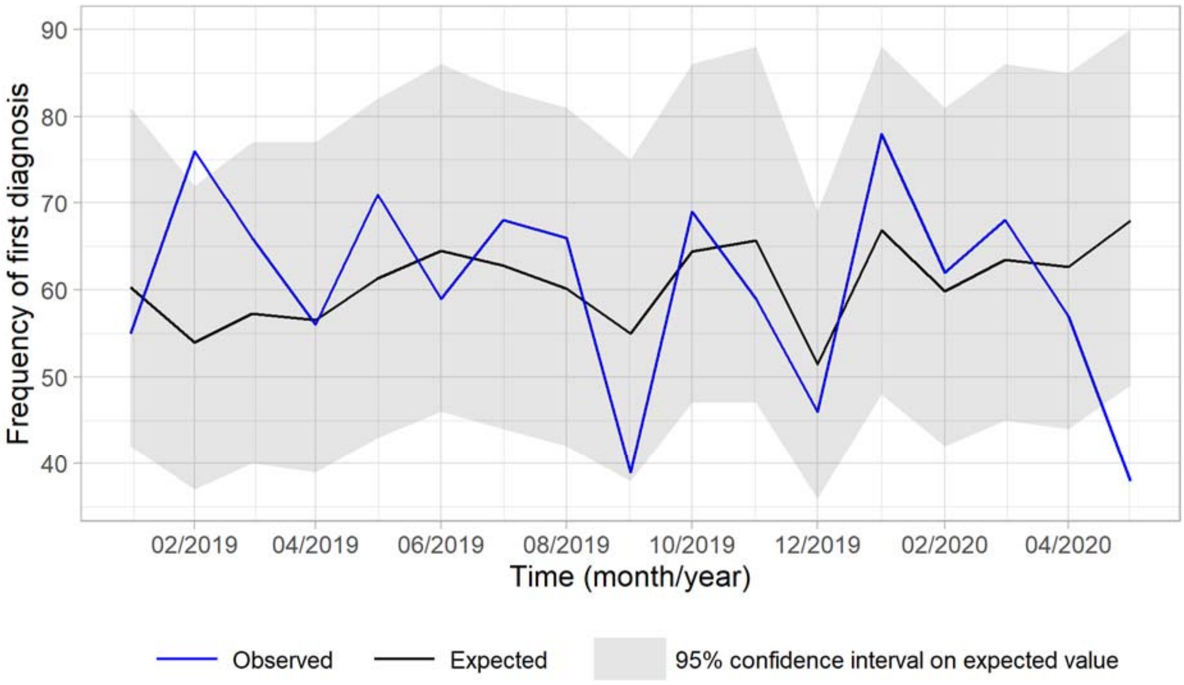

Figure S28 - First diagnosis of malignant cancer each month 2019-present. Expected line based on negative binomial regression model using data up to and including Feb 2020

Negative binomial regression tables

Table S1: The expected number of first diagnoses or first prescriptions as generated from the negative binomial regression models, and the number of first diagnoses and first prescriptions observed in the data, for March 2020. ACEIs - angiotensin-converting enzyme inhibitors, CCBs - calcium channel blockers, SSRIs - selective serotonin reuptake inhibitors.

**First Diagnosis / Prescription**	**Observed cases during March 2020**	**Expected cases between during March 2020 (95% CI)**	**Percentage reduction between the expected and observed cases during March 2020 (95% CI)**
Type II Diabetes	68	87 (59 to 124)	22·1% (-15·3% to 45·2%)
> Metformin	115	120 (91 to 153)	4·2% (-26·4% to 24·8%)
Circulatory system disease	278	362 (294 to 437)	23·2% (5·4% to 36·4%)
> Aspirin 75mg	99	105 (81 to 133)	6·0% (-22·2% to 25·6%)
> CCBs	197	217 (180 to 258)	9·1% (-9·4% to 23·6%)
> ACEIs	145	188 (150 to 229)	23·1% (3·3% to 36·7%)
> Clopidogrel	61	91 (63 to 128)	33·3% (3·2% to 52·3%)
Common mental health problems	519	771 (652 to 896)	32·6% (20·4% to 42·1%)
> SSRIs	212	260 (212 to 318)	18·5% (0·0% to 33·3%)
Malignant cancer	68	63 (45 to 86)	-7·2% (-51·1% to 20·9%)

Table S2: The expected number of first diagnoses or first prescriptions as generated from the negative binomial regression models, and the number of first diagnoses and first prescriptions observed in the data, for April 2020. ACEIs - angiotensin-converting enzyme inhibitors, CCBs - calcium channel blockers, SSRIs - selective serotonin reuptake inhibitors.

**First Diagnosis / Prescription**	**Observed cases during April 2020**	**Expected cases between during April 2020 (95% CI)**	**Percentage reduction between the expected and observed cases during April 2020 (95% CI)**
Type II Diabetes	41	97 (65 to 132)	57·5% (36·9% to 68·9%)
> Metformin	59	106 (79 to 137)	44·5% (25·3% to 56·9%)
Circulatory system disease	157	344 (275 to 419)	54·4% (42·9% to 62·5%)
> Aspirin 75mg	57	95 (72 to 119)	39·8% (20·8% to 52·1%)
> CCBs	87	173 (141 to 210)	49·8% (38·3% to 58·6%)
> ACEIs	48	163 (129 to 200)	70·6% (62·8% to 76·0%)
> Clopidogrel	56	81 (55 to 111)	30·5% (-1·8% to 49·5%)
Common mental health problems	266	666 (568 to 785)	60·1% (53·2% to 66·1%)
> SSRIs	114	231 (186 to 283)	50·6% (38·7% to 59·7%)
Malignant cancer	57	63 (44 to 85)	9·0% (-29·5% to 32·9%)

Table S3: The expected number of first diagnoses or first prescriptions as generated from the negative binomial regression models, and the number of first diagnoses and first prescriptions observed in the data, for May 2020. ACEIs - angiotensin-converting enzyme inhibitors, CCBs - calcium channel blockers, SSRIs - selective serotonin reuptake inhibitors.

First Diagnosis / Prescription	Observed cases during May 2020	Expected cases between during May 2020 (95% CI)	Percentage reduction between the expected and observed cases during May 2020 (95% CI)
Type II Diabetes	32	92 (62 to 127)	65·4% (48·4% to 74·8%)
> Metformin	39	105 (79 to 136)	62·8% (50·6% to 71·3%)
Circulatory system disease	163	348 (276 to 424)	53·1% (40·9% to 61·6%)
> Aspirin 75mg	57	101 (77 to 128)	43·7% (26·0% to 55·5%)
> CCBs	75	168 (135 to 202)	55·2% (44·4% to 62·9%)
> ACEIs	56	167 (132 to 204)	66·4% (57·6% to 72·5%)
> Clopidogrel	31	93 (64 to 128)	66·7% (51·6% to 75·8%)
Common mental health problems	288	711 (605 to 831)	59·5% (52·4% to 65·3%)
> SSRIs	123	246 (199 to 298)	50·1% (38·2% to 58·7%)
Malignant cancer	38	68 (49 to 90)	44·1% (22·4% to 57·8%)

Negative binomial model validation

To validate the negative binomial model we generated a model for data between January 2010 and February 2018. We then used this to predict the values for March - May 2018, to confirm that the observed values fell within the prediction confidence interval of the model. We repeated this for 2019. In both cases, all the observed values fell within the prediction confidence intervals as shown in the following tables

Table S4: The expected number of first diagnoses or first prescriptions as generated from negative binomial regression models fitted on data from January 2010 to February 2018, and the number of first diagnoses and first prescriptions observed in the data, for March to May 2018. ACEIs - angiotensin-converting enzyme inhibitors, CCBs - calcium channel blockers, SSRIs - selective serotonin reuptake inhibitors.

First Diagnosis / Prescription	Observed cases between March – May 2018	Expected cases between March – May 2018 (95% CI)	Percentage reduction between the expected and observed cases between March - May 2018 (95% CI)
Type II Diabetes	303	220 (143 to 313)	-37·8% (-111·9% to 3·2%)
> Metformin	311	289 (205 to 381)	-7·8% (-51·7% to 18·4%)
Circulatory system disease	918	880 (701 to 1080)	-4·3% (-31·0% to 15·0%)
> Aspirin 75mg	302	284 (217 to 358)	-6·4% (-39·2% to 15·6%)
> CCBs	486	489 (397 to 592)	0·7% (-22·4% to 17·9%)
> ACEIs	519	466 (365 to 578)	-11·5% (-42·2% to 10·2%)
> Clopidogrel	219	227 (156 to 312)	3·7% (-40·4% to 29·8%)
Common mental health problems	1846	1763 (1512 to 2041)	-4·7% (-22·1% to 9·6%)
> SSRIs	618	603 (481 to 738)	-2·5% (-28·5% to 16·3%)
Malignant cancer	177	140 (97 to 188)	-26·2% (-82·5% to 5·9%)

Table S5: The expected number of first diagnoses or first prescriptions as generated from negative binomial regression models fitted on data from January 2010 to February 2019, and the number of first diagnoses and first prescriptions observed in the data, for March to May 2019. ACEIs - angiotensin-converting enzyme inhibitors, CCBs - calcium channel blockers, SSRIs - selective serotonin reuptake inhibitors.

First Diagnosis / Prescription	Observed cases between March – May 2019	Expected cases between March – May 2019 (95% CI)	Percentage reduction between the expected and observed cases between March - May 2019 (95% CI)
Type II Diabetes	267	260 (171 to 364)	-2·7% (-56·1% to 26·6%)
> Metformin	287	317 (239 to 407)	9·5% (-20·1% to 29·5%)
Circulatory system disease	1068	961 (772 to 1174)	-11·1% (-38·3% to 9·0%)
> Aspirin 75mg	295	297 (224 to 375)	0·6% (-31·7% to 21·3%)
> CCBs	571	518 (419 to 624)	-10·3% (-36·3% to 8·5%)
> ACEIs	538	494 (391 to 608)	-8·8% (-37·6% to 11·5%)
> Clopidogrel	214	252 (173 to 340)	15·1% (-23·7% to 37·1%)
Common mental health problems	2131	1939 (1652 to 2255)	-9·9% (-29·0% to 5·5%)
> SSRIs	719	669 (535 to 819)	-7·4% (-34·4% to 12·2%)
Malignant cancer	193	171 (117 to 232)	-13·1% (-65·0% to 16·8%)

The RECORD statement – checklist of items, extended from the STROBE statement, that should be reported in observational studies using routinely collected health data.

	Item No.	STROBE items	Location in manuscript where items are reported	RECORD items	Location in manuscript where items are reported
**Title and abstract**					
	1	(a) Indicate the study's design with a commonly used term in the title or the abstract (b) Provide in the abstract an informative and balanced summary of what was done and what was found	**Title and abstract.**<br><br>**The study was a retrospective cohort study, and this is included in the title.**	RECORD 1.1: The type of data used should be specified in the title or abstract. When possible, the name of the databases used should be included.<br><br>RECORD 1.2: If applicable, the geographic region and timeframe within which the study took place should be reported in the title or abstract.<br><br>RECORD 1.3: If linkage between databases was conducted for the study, this should be clearly stated in the title or abstract.	**1.1 Methods section of abstract. The database name (SIR) is not widely known, so it is sufficient to say that it is GP EHR records from Salford, UK.**<br>**1.2 Methods section of abstract**<br>**1.3 No linkage occurred**
**Introduction**					
Background rationale	2	Explain the scientific background and rationale for the investigation being reported	**Section titled "Background and significance"**		
Objectives	3	State specific objectives, including any prespecified hypotheses	**Last paragraph of the "Background and significance" section**		
**Methods**					
Study Design	4	Present key elements of study design early in the paper	**In addition to the title this is also explained in the "Data analysis" section of the Methods**		
Setting	5	Describe the setting, locations, and relevant dates, including periods of recruitment, exposure, follow-up, and data collection	**First paragraph of the "Data source" section of the Methods**		
Participants	6	*(a) Cohort study* - Give the eligibility criteria, and the sources and methods of selection of participants. Describe methods of follow-up<br>*Case-control study* - Give the eligibility criteria, and the sources and methods of	**First paragraph of the "Data analysis" section of the Methods**	RECORD 6.1: The methods of study population selection (such as codes or algorithms used to identify subjects) should be listed in detail. If this is not possible, an explanation should be provided.	**6.1 Last para of the "Data source" section explains the methodology we have used to develop**

		case ascertainment and control selection. Give the rationale for the choice of cases and controls<br>*Cross-sectional study* - Give the eligibility criteria, and the sources and methods of selection of participants<br><br>*(b) Cohort study* - For matched studies, give matching criteria and number of exposed and unexposed<br>*Case-control study* - For matched studies, give matching criteria and the number of controls per case		RECORD 6.2: Any validation studies of the codes or algorithms used to select the population should be referenced. If validation was conducted for this study and not published elsewhere, detailed methods and results should be provided.<br><br>RECORD 6.3: If the study involved linkage of databases, consider use of a flow diagram or other graphical display to demonstrate the data linkage process, including the number of individuals with linked data at each stage.	**the clinical code sets.**<br>**6.2 The term set methodology is cited in the last para of the "Data source" section. Validation was also conducted by two GPs which is described in the same place**<br>**6.3 No data linkage was required.**
Variables	7	Clearly define all outcomes, exposures, predictors, potential confounders, and effect modifiers. Give diagnostic criteria, if applicable.	**"Data Analysis" section of the Methods**	RECORD 7.1: A complete list of codes and algorithms used to classify exposures, outcomes, confounders, and effect modifiers should be provided. If these cannot be reported, an explanation should be provided.	**7.1 All clinical code sets are made publically available via a git repo as mentioned in the last line of the Methods section**
Data sources/ measurement	8	For each variable of interest, give sources of data and details of methods of assessment (measurement). Describe comparability of assessment methods if there is more than one group	**The section "Data source" in the Methods.**		
Bias	9	Describe any efforts to address potential sources of bias	**Last para of "Data sources" explains the use of prescription data to combat bias if clinicians were not recording diagnoses.**		
Study size	10	Explain how the study size was arrived at	**The entire population of a CCG was used as that was the data that we had available. There was no need to take a sample.**		
Quantitative variables	11	Explain how quantitative variables were handled in the analyses. If applicable, describe which groupings were chosen, and why	**Not applicable**		
Statistical methods	12	(a) Describe all statistical methods, including those used to control for confounding	**"Data analysis" section of the Methods.**		

		(b) Describe any methods used to examine subgroups and interactions<br>(c) Explain how missing data were addressed<br>(d) *Cohort study* - If applicable, explain how loss to follow-up was addressed<br>*Case-control study* - If applicable, explain how matching of cases and controls was addressed<br>*Cross-sectional study* - If applicable, describe analytical methods taking account of sampling strategy<br>(e) Describe any sensitivity analyses			
Data access and cleaning methods		..		RECORD 12.1: Authors should describe the extent to which the investigators had access to the database population used to create the study population.<br><br>RECORD 12.2: Authors should provide information on the data cleaning methods used in the study.	**12.1 The last line of acknowledgements (as per Lancet policy).**<br>**12.2 Minimal data cleaning was required due to the nature of the study. Details of using only the first diagnosis of each condition and first prescription of each medication are provided in the last para of the "Data Source"**
Linkage		..		RECORD 12.3: State whether the study included person-level, institutional-level, or other data linkage across two or more databases. The methods of linkage and methods of linkage quality evaluation should be provided.	**No linkage**
**Results**					
Participants	13	(a) Report the numbers of individuals at each stage of the study (*e.g.*, numbers potentially eligible, examined for eligibility, confirmed eligible, included in the study, completing follow-up, and analysed)<br>(b) Give reasons for non-participation at each stage.	**Not required, as the whole population of Salford was included.**	RECORD 13.1: Describe in detail the selection of the persons included in the study (*i.e.,* study population selection) including filtering based on data quality, data availability and linkage. The selection of included persons can be described in the text and/or by means of the study flow diagram.	**Not really applicable as we were studying diagnoses rather than people. However this is covered in the "Data**

		(c) Consider use of a flow diagram			**source" section and Table 1**
Descriptive data	14	(a) Give characteristics of study participants (*e.g.*, demographic, clinical, social) and information on exposures and potential confounders<br>(b) Indicate the number of participants with missing data for each variable of interest<br>(c) *Cohort study* - summarise follow-up time (*e.g.*, average and total amount)	**Not applicable**		
Outcome data	15	*Cohort study* - Report numbers of outcome events or summary measures over time<br>*Case-control study* - Report numbers in each exposure category, or summary measures of exposure<br>*Cross-sectional study* - Report numbers of outcome events or summary measures	**Figures 1-5, Table 2 – also supplementary material**		
Main results	16	(a) Give unadjusted estimates and, if applicable, confounder-adjusted estimates and their precision (e.g., 95% confidence interval). Make clear which confounders were adjusted for and why they were included<br>(b) Report category boundaries when continuous variables were categorized<br>(c) If relevant, consider translating estimates of relative risk into absolute risk for a meaningful time period	**Results**		
Other analyses	17	Report other analyses done—e.g., analyses of subgroups and interactions, and sensitivity analyses	**None undertaken**		
**Discussion**					
Key results	18	Summarise key results with reference to study objectives	**"Key findings" section**		
Limitations	19	Discuss limitations of the study, taking into account sources of potential bias or imprecision. Discuss both direction and magnitude of any potential bias	**"Strengths and limitations" section**	RECORD 19.1: Discuss the implications of using data that were not created or collected to answer the specific research question(s). Include discussion of misclassification bias, unmeasured confounding, missing data, and changing eligibility over time, as they pertain to the study being reported.	**Second para of "Strengths and limitations"**

Interpretation	20	Give a cautious overall interpretation of results considering objectives, limitations, multiplicity of analyses, results from similar studies, and other relevant evidence	**"Comparison with existing literature" section and to a lesser extent "Implications" section**		
Generalisability	21	Discuss the generalisability (external validity) of the study results	**"Implications" section**		
**Other Information**					
Funding	22	Give the source of funding and the role of the funders for the present study and, if applicable, for the original study on which the present article is based	**"Funding" in abstract**		
Accessibility of protocol, raw data, and programming code		..		RECORD 22.1: Authors should provide information on how to access any supplemental information such as the study protocol, raw data, or programming code.	**Supplementary material available from journal web page. All code and redacted data available in a github repo as detailed in last line of Methods.**

*Reference: Benchimol EI, Smeeth L, Guttmann A, Harron K, Moher D, Petersen I, Sørensen HT, von Elm E, Langan SM, the RECORD Working Committee. The REporting of studies Conducted using Observational Routinely-collected health Data (RECORD) Statement. *PLoS Medicine* 2015; in press.

*Checklist is protected under Creative Commons Attribution (CC BY) license.
